# Optimisation of Culture Conditions Enhances Antifungal Activity and Reshapes Extracellular Metabolite Profiles in *Trichoderma harzianum* BOL-12QD

**DOI:** 10.3390/microorganisms14061331

**Published:** 2026-06-13

**Authors:** Luis Apaza Ticona, María Teresa Alvarez-Aliaga

**Affiliations:** 1 Organic Chemistry Unit, Department of Chemistry in Pharmaceutical Sciences, Faculty of Pharmacy, University Complutense of Madrid, Plza. Ramón y Cajal s/n, 28040 Madrid, Spain; 2 Molecular Biochemistry Area, Instituto de Investigaciones Fármaco Bioquímicas, Universidad Mayor de San Andrés, Av. Saavedra No 2224, Zona Miraflores, La Paz 00067, Bolivia; mtalvarez@umsa.bo

**Keywords:** *Botrytis cinerea*, culture filtrates, co-cultivation, exometabolome, NMR fingerprinting, secondary metabolism

## Abstract

*Botrytis cinerea* is a major phytopathogenic fungus responsible for substantial economic losses in horticultural crops, underscoring the need for sustainable alternatives to synthetic fungicides. This study investigated the influence of physical, chemical and biological culture parameters on the antifungal activity of culture filtrates produced by *Trichoderma harzianum* BOL-12QD. Culture conditions were sequentially optimised by evaluating light-filter exposure, carbon and nitrogen source composition, potato ecotype selection, co-cultivation with *Botrytis cinerea*, and volatile-mediated interactions. Antifungal activity was assessed using mycelial growth inhibition assays against *Botrytis cinerea*. Among the individual factors, violet-filter illumination, a medium containing 5 g L^−1^ glucose and 250 g L^−1^ potato extract, the *Leke Pek’e* potato ecotype, ammonium nitrate as nitrogen source, and co-cultivation with *Botrytis cinerea* at 10^4^ conidia mL^−1^ produced the highest inhibitory effects. Sequential integration of these optimised conditions resulted in enhanced antifungal activity, reaching up to 62% inhibition. Volatile organic compounds produced by *Trichoderma harzianum* BOL-12QD exhibited only minimal antifungal activity under the conditions tested, suggesting that volatile-mediated antagonism plays a limited role in this system. In contrast, culture-dependent modulation of extracellular metabolite profiles was evidenced by comparative ^1^H NMR fingerprinting, which revealed condition-specific spectral differences, with the optimised treatment displaying a distinct metabolic signature relative to all other conditions. Cytotoxicity assays in murine peritoneal macrophages showed no significant reduction in cell viability at concentrations up to 200 μg mL^−1^. In vivo exposure to the optimised culture filtrate (250 mg kg^−1^ d^−1^ for 10 days) induced transient treatment-related clinical observations without mortality, indicating a need for further detailed toxicological characterisation. Overall, these findings demonstrate that the antifungal activity of *Trichoderma harzianum* BOL-12QD is strongly modulated by interacting environmental, nutritional and biological culture parameters. The results support the potential of optimised culture filtrates as a source of bioactive metabolites for biocontrol applications, while highlighting the importance of integrated biochemical and toxicological evaluation.

## 1. Introduction

*Botrytis cinerea* Pers. ex Fr. (*B. cinerea*) is one of the most economically important necrotrophic phytopathogens worldwide, characterised by a broad host range and a remarkable capacity to adapt to diverse agroecological conditions [1]. This filamentous ascomycete infects more than 200 plant species and is associated with annual economic losses estimated to exceed US$30 billion in horticultural and fruit production systems [2]. Its pathogenic success is largely attributed to its genetic plasticity, prolific conidial production, and an extensive repertoire of virulence factors that facilitate host colonisation and evasion of plant defence responses [3].

The infection process involves germination of airborne conidia and development of specialised penetration structures capable of breaching plant cuticles and cell walls [4]. During colonisation, *B. cinerea* secretes numerous hydrolytic enzymes, including cellulases and proteases, which degrade structural plant components, while simultaneously producing phytotoxic metabolites such as botrydial and botcinins, together with reactive oxygen species that promote host cell death and facilitate necrotrophic growth [5,6,7]. These mechanisms contribute to its aggressiveness, particularly under conditions of high humidity and moderate temperatures [8].

Management of grey mould has traditionally relied on synthetic fungicides, including dicarboximides, anilinopyrimidines, and phenylpyrroles [9]. However, extensive use of these compounds has favoured the emergence of resistant populations through mutations affecting fungicide target sites and efflux mechanisms, with resistance frequencies exceeding 50% in several agricultural regions [10]. In parallel, increasingly stringent regulations aimed at reducing pesticide residues in agricultural products, including Regulation (EU) 2019/1793, have intensified the search for more sustainable and environmentally compatible alternatives [11].

Among the most promising biological control agents, species of the genus *Trichoderma* have attracted considerable attention due to their ability to suppress a wide range of phytopathogens through multiple complementary mechanisms [12]. These fungi combine mycoparasitism, competition for nutrients and ecological niches, secretion of hydrolytic enzymes, production of antifungal metabolites, induction of plant defence responses, and promotion of plant growth [13]. During mycoparasitism, pathogen-derived signals trigger chemotropic growth towards the host fungus, followed by adhesion, hyphal coiling, and secretion of chitinases, *β*-1,3-glucanases, and proteases that degrade fungal cell walls [14,15].

Among them, *Trichoderma harzianum* (*T. harzianum*) is one of the most extensively studied species owing to its broad-spectrum antagonistic activity against fungal pathogens, nematodes, and certain phytophagous insects [16]. Its efficacy results from the combined action of hydrolytic enzymes and a diverse repertoire of secondary metabolites, including peptaibols, polyketides, alkaloids, sesquiterpenes, and volatile organic compounds [16,17,18]. These metabolites disrupt membrane integrity, interfere with cellular metabolism, inhibit spore germination, and contribute to pathogen suppression. In addition, *T. harzianum* activates plant defence pathways mediated by jasmonic acid and salicylic acid, promoting the expression of defence-related genes (*PR-1*, *PR-5*, and *ACO*) and enhancing antioxidant responses, nutrient acquisition, and accumulation of protective secondary metabolites [19].

The biosynthesis of antifungal metabolites in *Trichoderma* is strongly influenced by environmental and nutritional conditions [20]. Factors such as nutrient availability, carbon-to-nitrogen ratio, light exposure, and microbial interactions regulate secondary metabolic pathways and determine both metabolite diversity and biological activity [20]. For example, violet light (400–480 nm) can stimulate *BLR-1*/*LOV* photoreceptor-mediated signalling pathways associated with conidiation and peptaibol production [21], whereas nitrogen availability and carbon-to-nitrogen balance influence the transition to the idiophase and accumulation of secondary metabolites [22]. Likewise, co-cultivation with phytopathogens may act as a biological elicitation strategy, inducing genes involved in antagonistic responses, including *prb1*, and increasing extracellular enzyme production [14].

Beyond their beneficial effects, some *Trichoderma* species are capable of producing secondary metabolites with potential toxicological relevance. Notably, certain strains have been reported to synthesise trichothecenes such as trichodermin, which inhibit eukaryotic protein synthesis and may induce oxidative stress [23,24]. These compounds have also been associated with phytotoxic effects, including reduced plant growth and root development [25]. Consequently, although *Trichoderma*-based biocontrol agents generally exhibit favourable safety profiles, secondary metabolite production should be assessed at the strain level, particularly when culture conditions are intentionally modified to enhance metabolite biosynthesis and biological activity [26,27].

Plant-derived substrates may further influence *Trichoderma* metabolism. In the Andean Altiplano, potato (*Solanum tuberosum*) ecotypes differ substantially in biochemical composition, including carbohydrate, protein, and phenolic contents, which may affect fungal growth and secondary metabolite biosynthesis [28,29]. Such variation offers opportunities to integrate local agrobiodiversity with microbial biotechnology and biological control strategies.

Despite the widespread commercial use of strains such as *T. harzianum* T-22 and *T. atroviride* IMI 206040, native strains adapted to specific agroecological environments remain comparatively understudied [30]. One example is *T. harzianum* BOL-12QD, a native Andean isolate that has previously demonstrated inhibitory activity of up to 54% against *B. cinerea* under controlled conditions [31]. Nevertheless, the environmental and nutritional factors governing its metabolite production, antifungal efficacy, and practical application remain insufficiently characterised [32].

Although numerous studies have demonstrated the biocontrol potential of *Trichoderma* species, many have focused on commercial strains, individual culture parameters, or conventional antagonism assays. Comparatively fewer studies have examined how sequential optimisation of culture conditions, biological elicitation, metabolite profiling, and functional evaluation of culture filtrates can be integrated to improve antifungal performance, particularly in native strains adapted to extreme agroecological environments. Furthermore, the relationship between culture-induced changes in metabolic profiles and antifungal activity remains incompletely understood.

Therefore, the present study evaluated the influence of selected physical, chemical, and biological culture conditions on the antifungal activity of *T. harzianum* BOL-12QD against *B. cinerea*. Culture parameters associated with enhanced activity were subsequently combined to obtain an optimised cultivation strategy, after which the resulting metabolite profile was characterised, and the biological activity of culture filtrates was assessed under controlled experimental conditions. By linking culture optimisation with metabolite profiling and antifungal evaluation, this work contributes to a better understanding of the factors influencing secondary metabolism in a native *Trichoderma* strain and provides a basis for the development of sustainable disease management strategies in Andean agricultural systems.

## 2. Materials and Methods

### 2.1. Fungal Strains

*Trichoderma harzianum* (GenBank accession number KY644517.1; strain BOL-12QD) and the phytopathogen *Botrytis cinerea* (Chirapaca 1) were used in this study. Both fungal strains were obtained from the culture collection of the Instituto de Investigaciones Farmaco-Bioquímicas (IIFB), La Paz, Bolivia, where they are maintained under standard preservation conditions for filamentous fungi.

The *T. harzianum*–*B. cinerea* system was selected as a biologically relevant model of antagonistic interaction because of the well-established role of *B. cinerea* as a major phytopathogen in Andean agricultural systems and the recognised use of *T. harzianum* as a biocontrol agent [33,34].

All experiments were initiated using freshly reactivated subcultures derived from actively growing stock cultures maintained in the institutional collection. These stock cultures were periodically subcultured under identical conditions to preserve physiological stability and minimise variability associated with long-term storage.

For each experimental assay, inocula were prepared from freshly reactivated subcultures to ensure biological consistency across experiments. Each independent biological replicate was defined as an assay initiated from an independently reactivated subculture derived from the corresponding stock culture rather than as repeated measurements obtained from the same culture.

All independent subcultures were generated under identical growth conditions to ensure physiological comparability while maintaining experimental independence among replicates.

### 2.2. Activation and Maintenance of Fungal Cultures

Fungal strains were activated according to the method described by Tyśkiewicz et al. [35], allowing recovery of metabolic activity from preserved cultures and the establishment of genetically stable monosporic isolates.

Conidial suspensions were prepared in sterile distilled water using a Milli-Q^®^ Direct 8 system (18.2 MΩ·cm at 25 °C; Sigma-Aldrich, Cat. No. ZIQ7000T0C, St. Louis, MO, USA) and adjusted to concentrations of 1 × 10^3^, 1 × 10^4^, and 1 × 10^6^ conidia mL^−1^ for controlled serial dilution during the isolation procedure.

For monosporic culture establishment, five 20 µL droplets of sterile water were placed onto sporulating regions of potato dextrose agar (PDA; Sigma-Aldrich, Cat. No. P2182, St. Louis, MO, USA). The droplets were examined under an optical microscope equipped with a planachromatic 40×/0.65 objective (Olympus BX53; Olympus Corporation, Tokyo, Japan), and those containing suitable conidial densities were selected for single-spore isolation.

The selected suspension was inoculated onto the centre of PDA plates supplemented with chloramphenicol (≥98%, Sigma-Aldrich, CAS No. 56-75-7, St. Louis, MO, USA; 75 µg mL^−1^) using a sterile inoculation loop to suppress bacterial contamination.

The plates were incubated at 25 ± 1 °C and 85% relative humidity in a controlled-environment incubator (Memmert INE501, 161 L capacity, temperature precision ± 0.1 °C; Memmert GmbH, Schwabach, Germany) until complete colony development (7–10 d). Subsequently, hyphal tip isolation was carried out by excising 6 mm agar plugs from the actively growing colony margins using a sterile cork borer.

Individual hyphal tips were selected under a stereomicroscope (Olympus SZ61, magnification 10–67×, field of view 22 mm; Olympus Corporation, Tokyo, Japan) and transferred onto fresh PDA plates supplemented with chloramphenicol (75 µg mL^−1^). The plates were then incubated at 25 ± 1 °C for 5–7 d until morphologically uniform pure cultures were obtained.

Each independent biological replicate was defined as a distinct culture initiated from an independently prepared monosporic isolate generated through separate inoculation events under identical culture conditions rather than as a technical replicate or repeated sampling from the same fungal culture.

Culture filtrates used in subsequent experiments were obtained independently from each biological replicate, ensuring that each experimental unit represented a biologically independent culture rather than repeated measurements derived from a single culture system.

### 2.3. Morphological Characterisation and Taxonomic Identification

Morphological characterisation of purified cultures was conducted through both macroscopic and microscopic analyses. For macroscopic evaluation, 7-day-old cultures grown on potato dextrose agar (PDA; Sigma-Aldrich, Cat. No. P2182, St. Louis, MO, USA) supplemented with chloramphenicol (≥98%, Sigma-Aldrich, CAS No. 56-75-7, St. Louis, MO, USA; 75 µg mL^−1^) were cultivated in 90 mm Petri dishes. Colony diameter was determined using a digital calliper.

Macroscopic characteristics, including colony texture (cottony, velvety, powdery, or compact), reverse pigmentation, and the presence of exudates, were documented by digital photography using a Canon EOS 90D camera fitted with an EF-S 60 mm f/2.8 USM macro lens (Canon Inc., Tokyo, Japan). Colony pigmentation was further quantified with a Konica Minolta CM-26d spectrophotometer (d/8° geometry; Konica Minolta, Inc., Tokyo, Japan) under standardised conditions.

All macroscopic measurements were obtained from independent biological replicate cultures, each originating from separately established fungal cultures. Multiple technical measurements were recorded for each plate to ensure measurement consistency.

For microscopic characterisation, mycelial fragments were prepared using the adhesive tape technique and mounted on microscope slides. The preparations were stained with 1% (*w*/*v*) methylene blue (≥82%, Sigma-Aldrich, CAS No. 122965-43-9, St. Louis, MO, USA) and examined using an Olympus BX53 microscope equipped with planachromatic objectives (10×/0.25 and 40×/0.75; Olympus Corporation, Tokyo, Japan).

To preserve the native three-dimensional architecture of the reproductive structures, a slide culture technique was also employed. Sterile coverslips were placed on sterile filter paper moistened with sterile distilled water, and conidial suspensions (1 × 10^6^ conidia mL^−1^) were inoculated onto the coverslips before incubation for 5–7 d at 25 ± 1 °C. The coverslips were subsequently mounted in lactophenol cotton blue (Sigma-Aldrich, Cat. No. 61335, St. Louis, MO, USA) and examined under oil immersion using an Olympus BX53 microscope at 100× magnification.

Taxonomic identification was based on the morphological criteria described by Barnett and Hunter [36], including conidial morphology (shape, size, septation, and arrangement), organisation of reproductive structures, hyphal characteristics, and colony morphology. The final identification was established through integration of the macroscopic and microscopic characteristics in accordance with established taxonomic keys for filamentous fungi.

### 2.4. Conidial Quantification and Inoculum Standardisation

Following morphological confirmation of fungal identity, conidial quantification was carried out to standardise inocula for subsequent experiments. Fungal colonies were harvested using a sterile spatula and resuspended in sterile distilled water. The resulting suspension was filtered through Miracloth (Calbiochem, Cat. No. 475855, La Jolla, CA, USA) to remove mycelial fragments and obtain a homogeneous preparation suitable for enumeration. Aliquots (10 µL) of the homogenised suspension were loaded into a Neubauer haemocytometer and covered with a coverslip, ensuring complete filling of the chamber without air bubble formation.

Conidia were enumerated under phase-contrast microscopy using an Olympus BX53 microscope equipped with a planachromatic objective (UPLXAPO 40×/0.75; Olympus Corporation, Tokyo, Japan). The conidia present in the four corner squares of the haemocytometer were counted, and the final concentration was calculated according to the standard haemocytometer equation:

Conidia mL^−1^ = (number of counted conidia × dilution factor × 10^4^)/number of squares counted.

All conidial suspensions were prepared from independently established biological replicates, ensuring that each quantified suspension represented an independent biological unit.

For each biological replicate, conidial concentration was determined from three independent haemocytometer counts obtained using separately loaded chambers, thereby distinguishing technical variability from biological variability.

This procedure enabled accurate standardisation of inoculum concentrations for subsequent experimental assays, ranging from 1 × 10^3^ to 1 × 10^6^ conidia mL^−1^.

### 2.5. Culture Optimisation Strategy for T. harzianum BOL-12QD

To maximise the production of biologically active secondary metabolites, the influence of chemical, physical, and biological in vitro culture conditions on fungal growth and metabolic activity was evaluated in *T. harzianum* BOL-12QD through a structured sequential optimisation strategy.

The optimisation strategy involved the systematic assessment of individual variables under controlled conditions while maintaining all non-tested parameters constant within each experimental set. This approach enabled independent evaluation of physical (e.g., light exposure), chemical (e.g., carbon and nitrogen sources), and biological (pathogen presence) factors influencing fungal performance.

Each condition was established using independently prepared fungal cultures, which served as the biological experimental units. Accordingly, each treatment represented a distinct culture system initiated de novo under identical baseline conditions, thereby ensuring independence among experimental sets.

Importantly, this sequential optimisation strategy did not constitute a multifactorial experimental design; therefore, interaction effects among variables were not evaluated within this framework.

All experiments were conducted under aseptic conditions in a laminar flow hood (Telstar Bio II Advance; Telstar, Terrassa, Barcelona, Spain). Sterile distilled water obtained from a Milli-Q^®^ Direct 8 system (18.2 MΩ·cm at 25 °C; Sigma-Aldrich, Cat. No. ZIQ7000T0C, St. Louis, MO, USA) was used throughout the study to ensure reproducibility and minimise external contamination.

#### 2.5.1. Definition of Biological and Technical Replicates

Independent biological replicates were defined as separate fungal cultures established from independent inoculation events originating from the monosporic stock culture. Each biological replicate consisted of an independently prepared culture flask, inoculated separately and incubated under identical conditions, with every culture processed independently throughout all subsequent analyses. Accordingly, the biological replicate constituted the experimental unit for all assays.

Technical replicates consisted of repeated measurements obtained from the same biological replicate and were included to assess analytical precision and measurement variability.

Time-course sampling involved repeated measurements collected from the same biological replicate over time to monitor dynamic changes in culture behaviour. These temporal observations do not constitute independent biological replicates and were therefore not treated as independent samples in the statistical analyses, as they originated from a single experimental unit.

#### 2.5.2. Physical Culture Conditions

To investigate the influence of light quality on the production of bioactive metabolites, *T. harzianum* BOL-12QD was cultivated in potato dextrose broth (PDB; Sigma-Aldrich, Cat. No. P6685, St. Louis, MO, USA) using 250 mL Erlenmeyer flasks containing 100 mL of medium. Each flask was inoculated independently with 100 µL of a conidial suspension (1 × 10^6^ conidia mL^−1^), thereby ensuring a standardised initial inoculum across all treatments. Each flask constituted one independent biological replicate.

The cultures were exposed to different illumination regimes using coloured cellophane films to generate white, violet, blue, green, and red light-filtered environments, whereas complete darkness was achieved by double wrapping the flasks with aluminium foil. Although these filters provided operational colour-filtered illumination, the transmitted spectral distribution, wavelength range, photon flux density, and absolute light intensity were not experimentally quantified. Consequently, the cultures were maintained under the ambient incubator illumination regime, and the treatments should be interpreted as colour-filtered light environments rather than precisely wavelength-defined exposures. Therefore, conclusions regarding spectral-specific responses should be regarded as exploratory.

The flasks were sealed with cotton plugs and aluminium foil to minimise contamination and evaporation while allowing gas exchange. Cultures were incubated at 25 ± 1 °C under static conditions for 30 d in a temperature-controlled incubator (Memmert IN55; Memmert GmbH, Schwabach, Germany).

To monitor temporal changes in extracellular metabolic activity, 1 mL aliquots of culture supernatant were aseptically collected from each flask at 24 h intervals. The samples were centrifuged at 3000 rpm for 10 min (Eppendorf 5804R; Eppendorf AG, Hamburg, Germany), and the clarified supernatants were stored in sterile microtubes at −20 °C until evaluation of antifungal activity against *B. cinerea* (1 × 10^6^ conidia mL^−1^).

Because each flask represented an independent biological replicate, repeated sampling from the same flask over time constituted repeated measurements of a single experimental unit and was therefore not considered independent biological replication in the statistical analyses.

#### 2.5.3. Nutritional Parameters Optimisation

##### Carbon Source Evaluation System

To identify the most suitable culture medium for secondary metabolite biosynthesis, a 3^2^ factorial design was employed based on different concentrations of potato extract and glucose.

Fresh high Andean potatoes (*Solanum tuberosum* subsp. *andigena*) were washed, peeled, and cut into approximately 2 cm^3^ pieces before being boiled in distilled water for 20 min. The resulting extract was filtered through sterile gauze, allowed to cool under aseptic conditions, and adjusted to the required final volume.

The experimental design included three levels of potato extract (62.5, 125, and 250 g L^−1^) combined with three levels of *D*-(+)-glucose anhydrous (≥99.5%, Sigma-Aldrich, CAS No. 50-99-7, St. Louis, MO, USA; 2, 5, and 10 g L^−1^), according to a full 3^2^ factorial design. Each treatment was established using independently prepared culture flasks, with each flask representing one independent biological replicate.

Before sterilisation, the medium pH was adjusted to 6.0 using sterile hydrochloric acid (HCl, 37%, Sigma-Aldrich, CAS No. 7647-01-0, St. Louis, MO, USA) or sodium hydroxide (NaOH, ≥97.0%, pellets, Sigma-Aldrich, CAS No. 1310-73-2, St. Louis, MO, USA). The media were subsequently sterilised by autoclaving (Raypa AES 75, Barcelona, Spain) at 121 °C for 15 min.

Each flask containing 100 mL of medium was inoculated independently with 100 µL of a fresh conidial suspension (1 × 10^6^ conidia mL^−1^) and incubated under static conditions at 25 °C in partial darkness for 30 d. Each flask constituted an independent biological replicate.

To monitor extracellular metabolite production, 1 mL aliquots of culture supernatant were aseptically collected from each flask at predetermined time intervals. The samples were centrifuged to remove cellular debris, and the resulting supernatants were stored at −20 °C until antifungal activity against *B. cinerea* was evaluated. Repeated samples obtained from the same culture flask over time were not regarded as independent biological replicates and were therefore excluded from the assumption of statistical independence.

##### Potato Ecotype Variation Assessment

To evaluate the influence of potato genetic variability on metabolite production, culture media were prepared using different Andean potato ecotypes, including *Condor Imilla* (*Solanum tuberosum* subsp. *andigena*), *Yana Runa* (*Solanum tuberosum* subsp. *andigena*), *Leke Pek’e* (*Solanum tuberosum* subsp. *andigena*), and *Luk’i Turno* (*Solanum* × *juzepczukii*), together with the commercial cultivar *Dutch Désirée* (*Solanum tuberosum*), purchased from Ketal supermarket. All Andean ecotypes were taxonomically identified and authenticated by Fundación PROINPA (Promoción e Investigación de Productos Andinos).

All media were prepared according to the extraction protocol and standardised conditions described in Section Carbon Source Optimisation, thereby ensuring consistent processing, pH adjustment, sterilisation, and baseline nutrient composition across all ecotypes.

For each ecotype, biological replicates consisted of separately prepared culture flasks, each inoculated independently with a fresh conidial suspension derived from the monosporic stock culture. This approach ensured a constant inoculum source across all treatments, while biological replication was achieved through independent culture preparation. Each flask received 100 µL of a 1 × 10^6^ conidia mL^−1^ suspension and was incubated under identical temperature and static culture conditions.

Culture supernatants were collected daily from each flask under aseptic conditions, centrifuged to remove cellular debris, and stored at −20 °C until antifungal activity against *B. cinerea* was evaluated. These longitudinal samples represented repeated measurements from the same biological replicate and were therefore not treated as independent observations in the statistical analyses.

##### Nitrogen Source Optimisation

Four nitrogen sources were evaluated to determine their influence on secondary metabolite biosynthesis. A basal medium was prepared containing 39 g L^−1^ potato dextrose agar (PDA; Sigma-Aldrich, Cat. No. P2182, St. Louis, MO, USA), supplemented with 1 g L^−1^ magnesium sulphate heptahydrate (MgSO_4_·7H_2_O, ≥98%, Sigma-Aldrich, CAS No. 10034-99-8, St. Louis, MO, USA), 1 g L^−1^ monopotassium phosphate (KH_2_PO_4_, ≥99.0%, Sigma-Aldrich, CAS No. 7778-77-0, St. Louis, MO, USA), 1 g L^−1^ ammonium sulphate ((NH_4_)_2_SO_4_, ≥99%, Sigma-Aldrich, CAS No. 7783-20-2, St. Louis, MO, USA), and 1 g L^−1^ fosetyl-aluminium (Fosetyl-Al, Fisher Scientific, Cat. No. 17201196, Madrid, Spain). In addition, 0.3 g L^−1^ of an antibiotic mixture comprising gentamicin sulphate (≥590 IU, Sigma-Aldrich, CAS No. 1405-41-0, St. Louis, MO, USA) and tetracycline hydrochloride (≥95%, Sigma-Aldrich, CAS No. 64-75-5, St. Louis, MO, USA) was included to prevent bacterial contamination during prolonged incubation.

Fosetyl-Al was incorporated as a supplementary organophosphonate component of the basal medium rather than serving as a nitrogen source. Its concentration was kept constant across all treatments to preserve medium composition and enable evaluation of nitrogen-source-dependent responses under identical phosphonate-associated culture conditions [37].

The variable nitrogen sources consisted of bacteriological peptone (Sigma-Aldrich, Cat. No. 1.07228, St. Louis, MO, USA), urea (CO(NH_2_)_2_, ≥99%, Sigma-Aldrich, CAS No. 57-13-6, St. Louis, MO, USA), ammonium nitrate (NH_4_NO_3_, ≥98%, Sigma-Aldrich, CAS No. 6484-52-2, St. Louis, MO, USA), and ammonium chloride (NH_4_Cl, ≥99.5%, Sigma-Aldrich, CAS No. 12125-02-9, St. Louis, MO, USA). Each was incorporated individually into the basal medium, replacing equivalent nitrogen contributions to ensure comparability among treatments.

For each nitrogen source, biological replicates consisted of separately prepared culture flasks, each representing a distinct experimental unit inoculated independently with a fresh conidial suspension derived from the monosporic stock culture. This approach ensured that variability was captured at the level of culture preparation rather than within a single culture system. Each flask received an inoculum of 1 × 10^6^ conidia mL^−1^ and was incubated at 25 °C under static conditions for 30 d.

Culture supernatants were collected daily from each biological replicate under aseptic conditions, centrifuged to remove cellular debris, and stored at −20 °C until antifungal activity against *B. cinerea* was evaluated. These longitudinal samples represented repeated measurements of the same biological system and were therefore not considered independent biological replicates in the statistical analyses.

#### 2.5.4. Biological Interactions

##### Co-Culture System with *B. cinerea*

To evaluate the role of direct biological interactions in the induction of inhibitory metabolites, co-culture assays were conducted using *T. harzianum* BOL-12QD and *B. cinerea* in liquid potato dextrose broth (PDB; Sigma-Aldrich, Cat. No. P6685, St. Louis, MO, USA).

*T. harzianum* BOL-12QD was first inoculated into 100 mL of PDB at a final concentration of 1 × 10^6^ conidia mL^−1^ and incubated at 25 °C for 15 d under static conditions to establish biomass and stabilise metabolic activity within each culture system.

For each condition, biological replicates consisted of separately prepared culture flasks, each representing an independent co-culture system initiated from the monosporic stock culture. These flasks served as the experimental units for all downstream analyses.

Subsequently, *B. cinerea* (100 µL of 1 × 10^4^ conidia mL^−1^ suspension) was introduced into each pre-established *Trichoderma* culture, enabling direct fungal–fungal interaction within each biological replicate.

Co-cultures were incubated for a further 15 d under identical conditions. To monitor interaction-associated metabolite production over time, 1 mL aliquots of culture supernatant were aseptically collected at 24 h intervals from each biological replicate, centrifuged to remove cellular debris, and stored at −20 °C until antifungal activity assays.

These longitudinal samples represented repeated measurements of the same biological system and were therefore not considered independent biological replicates in the statistical analyses.

Because metabolites present in co-culture filtrates may originate from either fungal species or arise from interaction-induced physiological responses, subsequent analyses focused on the overall biological activity of the co-culture system rather than attribution of metabolite origin to a specific organism.

##### Culture Filtrates for Self-Inhibitory Assays

Control cultures of *B. cinerea* were established under identical conditions to those described for the co-culture system to define baseline growth and metabolic activity.

Briefly, 100 µL of a *B. cinerea* conidial suspension (1 × 10^6^ conidia mL^−1^) was inoculated into 100 mL of potato dextrose broth (PDB; Sigma-Aldrich, Cat. No. P6685, St. Louis, MO, USA). For each condition, biological replicates consisted of separately prepared culture flasks, each representing an independent experimental unit initiated from the monosporic stock culture.

Cultures were incubated at 25 °C under static conditions for 30 d in the absence of *Trichoderma*, thereby providing a reference system reflecting intrinsic fungal growth and metabolic behaviour.

Culture supernatants were collected daily under aseptic conditions from each biological replicate, centrifuged to remove cellular debris, and stored at −20 °C until analysis. These longitudinal samples represented repeated measurements of the same biological system and were therefore not considered independent for statistical analysis.

The resulting filtrates served as baseline references for comparison with co-culture-derived supernatants under a matched experimental design, ensuring system-level comparability between treatments.

##### Volatile Organic Compound (VOC) Interaction Assay

The production and biological activity of volatile organic compounds (VOCs) emitted by *T. harzianum* BOL-12QD were evaluated using a paired-flask system connected by a sterile glass tube (10–15 cm in length), enabling a shared headspace while preventing any physical contact or liquid-phase exchange between cultures.

For each condition, biological replicates consisted of separately assembled paired-flask systems, each constructed independently from monosporic stock cultures. Each paired configuration, comprising one *T. harzianum* flask and one *B. cinerea* flask connected via a shared headspace, was considered a single biological unit for statistical analysis.

In each system, the *T. harzianum* flask (100 µL of 1 × 10^6^ conidia mL^−1^ suspension) was incubated for 48 h to establish baseline metabolic activity and VOC emission. Subsequently, a second flask containing *B. cinerea* (100 µL of 1 × 10^6^ conidia mL^−1^ suspension) was introduced into the system, allowing exposure to fungal volatiles in a physically separated configuration.

The assembled paired systems were sealed with Parafilm M^®^ (Amcor Flexibles North America, Inc., Houston, TX, USA) to maintain headspace integrity and incubated at 25 °C under static conditions for 15 d.

Radial growth of *B. cinerea* was measured daily along eight equidistant axes from the inoculation point. The mean of these measurements was used as a single technical estimate of colony diameter for each biological replicate (paired system), ensuring spatial consistency in growth assessment.

Growth modulation, expressed as inhibition or stimulation, was calculated relative to monoculture and paired control systems. Control systems consisted of identical paired-flask assemblies in which *B. cinerea* was exposed to sterile, uninoculated medium instead of *T. harzianum*, thereby enabling discrimination between VOC-mediated effects and baseline growth dynamics under an identical experimental architecture.

### 2.6. In Vitro Antifungal Activity and Fungal Growth Kinetics Assays

To evaluate the antifungal activity of *T. harzianum* BOL-12QD culture filtrates against *B. cinerea*, two complementary in vitro assays were performed: radial growth kinetics and a disc diffusion inhibition assay. Together, these approaches provided quantitative and spatial resolution of fungal growth responses under treatment conditions.

For growth kinetics analysis, 100 µL of a *B. cinerea* conidial suspension (1 × 10^5^ conidia mL^−1^) were inoculated at the centre of Petri dishes containing potato dextrose agar (PDA; Sigma-Aldrich, Cat. No. P2182, St. Louis, MO, USA). Plates were incubated at 25 ± 1 °C, and radial colony expansion was monitored daily for up to 7 d.

To account for anisotropic mycelial growth, colony radius was measured along eight equidistant axes (0°, 45°, 90°, 135°, 180°, 225°, 270°, and 315°) using a digital calliper (±0.01 mm resolution). Mean colony radius at each time point was calculated as the arithmetic mean of the eight directional measurements. Radial growth rate was obtained from the slope of a linear regression fitted to mean colony radius over time (mm d^−1^), providing a quantitative descriptor of fungal expansion dynamics under each treatment.

For antifungal assessment using the diffusion-based inhibition assay, plates were uniformly surface-inoculated with a *B. cinerea* conidial suspension (1 × 10^5^ conidia mL^−1^) to generate a confluent mycelial lawn. Sterile paper discs (6 mm diameter) were impregnated with 100 µL of cell-free culture filtrate and placed on the agar surface, allowing radial diffusion of bioactive metabolites through the fungal lawn.

A commercial fungicide (chlorothalonil 50% *w*/*v*, 1:1000 dilution) was used as a positive control, while sterile distilled water served as a negative control. Plates were incubated at 25 ± 1 °C for 72 h.

Antifungal activity was quantified as inhibition halo diameter (mm), defined as the clear zone of growth suppression surrounding the disc, and as percentage mycelial growth inhibition, calculated using the equation:

%*I* = [(*C* − *T*)/*C*] × 100

where *C* represents the mean colony diameter of the control and *T* represents the mean colony diameter of treated samples.

Independent biological replicates corresponded to culture filtrates obtained from separately prepared fungal cultures, ensuring that each filtrate represented an independent biological experimental unit. Technical replicates consisted of repeated measurements performed on the same plate or filtrate to assess measurement precision. Each biological replicate was processed independently from culture preparation through antifungal testing.

### 2.7. ^1^H NMR-Based Metabolic Fingerprinting of Culture Filtrates

Following incubation under optimised conditions, fungal biomass was separated from the culture medium by centrifugation at 10,000× *g* for 15 min at 4 °C using a refrigerated Eppendorf 5810R centrifuge equipped with a fixed-angle F34-6-38 rotor (Eppendorf AG, Hamburg, Germany). The supernatant was sequentially clarified by gravity filtration through Whatman No. 1 filter paper and sterile filtration using Millex^®^ HV PVDF syringe filters (0.45 µm pore size, 33 mm; Sigma-Aldrich, Cat. No. SLHV033RS, St. Louis, MO, USA), yielding cell-free culture filtrates suitable for downstream metabolomic analysis.

Aliquots (100 mL) of the clarified filtrates were frozen at −80 °C and subsequently lyophilised under vacuum conditions (<0.1 mbar; condenser temperature approximately −55 °C) using a Christ Alpha 1-2 LDplus freeze dryer (Martin Christ GmbH, Osterode, Germany). The resulting dry residues were collected and weighed to determine total extract yield.

Lyophilised extracts were obtained from independently prepared fungal cultures under identical optimised conditions, each representing a biological replicate. Each replicate was processed independently throughout extraction and analytical workflows to maintain biological independence.

For metabolomic fingerprinting, lyophilised extracts were reconstituted in deuterated methanol (CD_3_OD, ≥99.8 atom% D; Sigma-Aldrich, CAS No. 811-98-3, St. Louis, MO, USA), homogenised, and transferred to 5 mm NMR tubes. Samples were analysed using a Bruker Avance III HD 500 MHz spectrometer equipped with a 5 mm TCI cryoprobe (Bruker BioSpin GmbH, Rheinstetten, Germany). ^1^H NMR spectra were acquired at 298 K using standardised parameters for metabolomic fingerprinting.

Spectral processing, including phase correction, baseline correction, peak alignment, and integration, was performed using MestReNova v.14.2.0 (Mestrelab Research S.L., Santiago de Compostela, Spain), ensuring consistency and reproducibility across samples.

Metabolomic analysis was restricted to comparative spectral fingerprinting across biological replicates. Accordingly, spectral signals were interpreted as reproducible metabolic features associated with each experimental condition, without assignment to specific chemical identities in the absence of authentic standards or LC–MS/MS confirmation. This strategy ensures that conclusions are based on robust pattern-level variation rather than tentative metabolite identification.

Therefore, all metabolomic interpretations are confined to comparative fingerprint-level differences among treatments and should not be interpreted as definitive evidence for the presence or absence of specific metabolite classes.

### 2.8. Biological Safety Assessment

#### 2.8.1. In Vitro Cytotoxicity Assay (MTT)

Cell viability was evaluated using the colorimetric MTT assay, based on the reduction of 3-(4,5-dimethylthiazol-2-yl)-2,5-diphenyltetrazolium bromide (MTT, ≥98%, Sigma-Aldrich, CAS No. 298-93-1, St. Louis, MO, USA) by mitochondrial succinate dehydrogenase, leading to the formation of insoluble formazan crystals.

Peritoneal macrophages were obtained from male Swiss mice (25–35 g, 6–8 weeks old; Charles River Laboratories), maintained under controlled environmental conditions (22 ± 2 °C, 12 h light/dark cycle) with ad libitum access to standard rodent chow and sterile distilled water prepared using a Milli-Q^®^ Direct 8 system (18.2 MΩ·cm at 25 °C; Sigma-Aldrich, Cat. No. ZIQ7000T0C, St. Louis, MO, USA).

All animal procedures were approved by the Ethics Committee of the Faculty of Veterinary Medicine, Universidad Complutense de Madrid (CEEA-UCM-2007-11-567) and authorised by the competent regional authority of the Community of Madrid, in accordance with Directive 2010/63/EU and Royal Decree 53/2013 governing animal experimentation.

Animals were anaesthetised by inhalation of ethyl ether (Et_2_O, ≥98.0%, Sigma-Aldrich, CAS No. 60-29-7, St. Louis, MO, USA) and humanely euthanised prior to cell collection. Peritoneal macrophages were harvested by intraperitoneal lavage with sterile phosphate-buffered saline (PBS, Sigma-Aldrich, Cat. No. P4474, St. Louis, MO, USA), followed by gentle abdominal massage and recovery of peritoneal exudate.

Cell suspensions were counted using a Neubauer haemocytometer, centrifuged at 1000 rpm for 10 min at 4 °C, and resuspended in RPMI 1640 medium (Sigma-Aldrich, Cat. No. R8758, St. Louis, MO, USA) supplemented with 10% foetal bovine serum (Sigma-Aldrich, Cat. No. F2442, St. Louis, MO, USA), 1% penicillin/streptomycin (Sigma-Aldrich, Cat. No. P4333, St. Louis, MO, USA), and 0.25 µg mL^−1^ amphotericin B (Sigma-Aldrich, CAS No. 1397-89-3, St. Louis, MO, USA). Final cell density was adjusted to 1 × 10^6^ cells mL^−1^.

A total of 5 × 10^5^ cells per well (200 µL) were seeded into 96-well plates and incubated for 18 h at 37 °C in a humidified atmosphere with 5% CO_2_ to allow adhesion and stabilisation.

Freeze-dried culture filtrates from optimised media were reconstituted in sterile distilled water and diluted in complete RPMI 1640 medium to final concentrations ranging from 0.1 to 100 µg mL^−1^. Cells were exposed to treatments for 3 h, after which MTT solution (0.5 mg mL^−1^) was added and incubation continued for a further 3 h. The supernatant was removed, and formazan crystals were solubilised in dimethyl sulfoxide (DMSO, ≥99.7%, Sigma-Aldrich, CAS No. 67-68-5, St. Louis, MO, USA).

Absorbance was measured at 580 nm using a microplate reader (BioTek Synergy^TM^ 2; BioTek Instruments, Winooski, VT, USA), and cell viability was expressed relative to untreated controls.

Cytotoxicity was evaluated using culture filtrates derived from independent biological replicates of *T. harzianum* BOL-12QD. Each biological replicate corresponded to an independently prepared fungal culture under optimised conditions. Technical replicates consisted of triplicate wells per condition within each biological replicate, with the biological replicate considered the experimental unit for statistical analysis.

#### 2.8.2. In Vivo Oral Toxicity Evaluation

The toxicological profile of the culture filtrate obtained from the optimised *T. harzianum* BOL-12QD culture medium was evaluated using a short-term oral exposure model in male Swiss mice (25–35 g; Charles River Laboratories, Barcelona, Spain).

Animals were housed individually in polypropylene cages under controlled environmental conditions (22 ± 2 °C, 55 ± 10% relative humidity, and a 12 h light/12 h dark cycle), with ad libitum access to standard rodent chow and sterile distilled water produced using a Milli-Q^®^ Direct 8 system (18.2 MΩ·cm at 25 °C; Sigma-Aldrich, Cat. No. ZIQ7000T0C, St. Louis, MO, USA).

After a 7-day acclimatisation period, animals were randomly assigned to four experimental groups (n = 5 per group): one control group and three treatment groups.

The culture filtrate was administered daily by oral gavage using a stainless-steel gastric probe at doses of 50, 100, and 250 mg kg^−1^ d^−1^. Dosing solutions were prepared in sterile distilled water and stabilised with 0.5% (*w*/*v*) gum Arabic (Sigma-Aldrich, CAS No. 9000-01-5, St. Louis, MO, USA) as a suspending agent, with a final administration volume of 10 mL kg^−1^ body weight.

Throughout the 10-day exposure period, animals were monitored daily for general health status, including body weight, food and water intake, and behavioural parameters such as spontaneous locomotor activity, posture, coat condition, tactile response, and signs of gastrointestinal disturbance.

Each animal constituted an independent experimental unit and was considered a biological replicate for statistical analysis. Culture filtrates used for administration were obtained from independently prepared *T. harzianum* BOL-12QD cultures grown under identical optimised conditions, ensuring batch-level biological independence. Animals were randomly allocated to experimental groups prior to exposure, and all observations were recorded at the level of individual animals without data pooling.

On day 10, animals were euthanised by intraperitoneal administration of sodium pentobarbital (150 mg kg^−1^; Sigma-Aldrich, CAS No. 57-33-0, St. Louis, MO, USA), followed by confirmation of death and macroscopic necropsy for gross examination of major organs.

### 2.9. Statistical Analysis

All experimental data were analysed using STATISTICA v.13.3 (TIBCO Software Inc., Palo Alto, CA, USA; academic licence; Intel^®^ Core^TM^ i7 processor, 32 GB RAM, Intel Corporation, Santa Clara, CA, USA). Group comparisons were performed using one-way analysis of variance (ANOVA), followed by Tukey’s honestly significant difference (HSD) post hoc test for multiple pairwise comparisons, where applicable.

Normality was assessed using the Shapiro–Wilk test, while homogeneity of variances was evaluated using Levene’s test. A significance threshold of α = 0.05 (95% confidence level) was applied throughout. Parametric tests were considered appropriate when assumptions of normality and homoscedasticity were satisfied.

Data are presented as mean ± standard deviation (SD). The number of biological replicates varied depending on the experimental system and is specified in each methodological section. In general, biological replication consisted of independently prepared experimental units, defined as either individual fungal culture batches or individual animals, depending on the assay.

Technical replicates were used to account for analytical variability and were averaged within each biological replicate prior to statistical analysis. Accordingly, all inferential analyses were performed at the level of biological replication to ensure independence of observations and avoid pseudoreplication.

For time-course experiments involving repeated sampling of the same biological replicate, data points were treated as repeated measurements of a single experimental unit and were not considered independent observations in inferential tests.

## 3. Results

### 3.1. Antifungal Activity of Culture Filtrates Under Different Culture Conditions

All graphical data representing *B. cinerea* growth are expressed as a percentage of the untreated control (control = 100%). For comparative purposes, antifungal activity is reported as percentage inhibition, calculated using the transformation: % inhibition = 100 − % relative growth.

This transformation was applied consistently across all experimental subsections (physical, chemical, and biological conditions) to ensure uniform interpretation of results. Statistical analyses were performed using the original growth data prior to transformation into inhibition values, thereby preserving the validity of inferential statistics.

#### 3.1.1. Effect of Light-Filter Conditions

**Figure 1:**
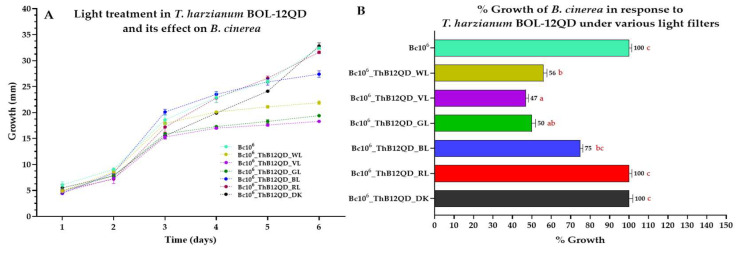
Effect of colour-filter (white = WL, violet = VL, green = GL, blue = BL, red = RL, and darkness = DK) culture conditions on the antifungal activity of *T. harzianum* BOL-12QD (ThB12QD) culture filtrates against *B. cinerea* (Bc10^6^; 10^6^ conidia mL^−1^). (**A**) Growth kinetics of *B. cinerea* exposed to culture filtrates obtained under different colour-filter conditions. (**B**) *B. cinerea* growth expressed as a percentage of the untreated control (control = 100%). Percentage inhibition values reported in the text were calculated as 100 − % relative growth. Values are expressed as mean ± SD (n = 5 biological replicates). Different letters indicate statistically significant differences among treatments (one-way ANOVA followed by Tukey’s HSD, *p* < 0.05).

The antifungal activity of culture filtrates from *T. harzianum* BOL-12QD, obtained under different colour-filter conditions, was evaluated based on their effects on *B. cinerea* growth. In Figure 1B, fungal growth is expressed as a percentage of the untreated control (control = 100%), while antifungal activity is reported in the text as percentage inhibition, calculated as 100 − % relative growth.

One-way ANOVA indicated that culture illumination significantly affected the antifungal activity of the resulting filtrates (F_4,10_ = 18.72; *p* < 0.0001). However, Tukey’s HSD post hoc analysis indicated that the magnitude of these differences was generally modest across treatments.

Among the tested conditions, violet-filter-derived filtrates produced the lowest relative growth of *B. cinerea* (47%), corresponding to 53% inhibition. This effect was significantly higher than that observed under the white-filter condition (56% growth; 44% inhibition; *p* = 0.008). Green-filter-derived filtrates resulted in 50% relative growth (50% inhibition), whereas blue-filter conditions yielded 75% relative growth (25% inhibition). No statistically significant differences were detected between green, white, and blue treatments in pairwise comparisons after correction for multiple testing.

In contrast, filtrates obtained under red-filter and dark conditions did not reduce fungal growth, with both conditions recording 100% relative growth (0% inhibition), indicating an absence of detectable antifungal activity.

Consistently, violet-filter-derived filtrates caused a slight reduction in radial growth rate of *B. cinerea* (5.3 mm d^−1^ vs. 5.5 mm d^−1^ in the control) and a marginal decrease in final colony diameter (15 mm vs. 18 mm). Although statistically significant, these differences indicate a limited biological effect size.

Overall, violet-filter conditions exhibited the highest antifungal activity among the tested treatments, although the magnitude of the effect remained moderate. Since spectral distribution and irradiance were not experimentally quantified, these treatments should be interpreted as operational light-filter conditions rather than defined wavelength-specific photobiological exposures.

#### 3.1.2. Effect of Nutritional Parameters

##### Carbon Source Composition

The influence of culture medium composition on the antifungal activity of *T. harzianum* BOL-12QD culture filtrates was evaluated by varying glucose concentrations (2, 5, and 10 g L^−1^) in combination with potato extract concentrations (62.5, 125, and 250 g L^−1^) using a factorial screening approach. Glucose served as a readily metabolisable carbon source, whereas potato extract provided a complex nutrient matrix enriched in starch and polysaccharides.

**Figure 2:**
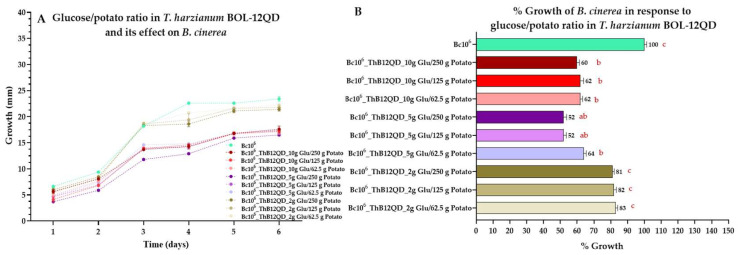
Effect of glucose and potato extract concentrations on the antifungal activity of *T. harzianum* BOL-12QD (ThB12QD) culture filtrates against *B. cinerea* (Bc10^6^; 10^6^ conidia mL^−1^). (**A**) Radial growth kinetics of *B. cinerea* exposed to culture filtrates obtained under different glucose and potato extract combinations. (**B**) *B. cinerea* growth expressed as a percentage of the untreated control (control = 100%). Percentage inhibition values reported in the text were calculated as 100 − % relative growth. Values are expressed as mean ± SD (n = 5 biological replicates). Different letters indicate statistically significant differences among treatments (one-way ANOVA followed by Tukey’s HSD, *p* < 0.05).

As illustrated in Figure 2B, fungal growth is expressed as a percentage of the untreated control (control = 100%), while antifungal activity is reported as percentage inhibition, calculated as 100 − % relative growth.

Statistical analysis indicated that culture medium composition significantly influenced the antifungal activity of the resulting filtrates (one-way ANOVA: F_8,18_ = 22.45; *p* < 0.0001).

The lowest *B. cinerea* growth was observed in media containing 5 g L^−1^ glucose combined with 125 or 250 g L^−1^ potato extract, both yielding identical values (52% relative growth; 48% inhibition). These treatments were statistically indistinguishable, indicating comparable antifungal performance.

Intermediate effects were observed at 10 g L^−1^ glucose, with relative growth ranging from 60–62% (38–40% inhibition) across potato extract concentrations. In contrast, low glucose conditions (2 g L^−1^) resulted in reduced activity, with 81–83% relative growth (17–19% inhibition).

Tukey’s HSD post hoc analysis confirmed that the 5 g L^−1^ glucose treatments were significantly more effective than most other combinations (*p* < 0.05), whereas differences among intermediate and low-activity groups were less pronounced.

Overall, antifungal activity was strongly influenced by carbon source composition, with intermediate glucose concentrations consistently associated with higher inhibition. Based on radial growth kinetics (Figure 2A), the condition 5 g L^−1^ glucose + 250 g L^−1^ potato extract was selected for subsequent experiments due to sustained reduction in colony expansion and smaller final colony diameter over time, despite being equivalent in endpoint inhibition to the 125 g L^−1^ potato extract condition.

##### Potato Ecotypes Selection

**Figure 3:**
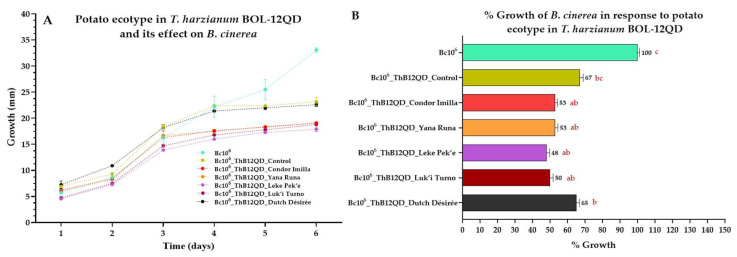
Effect of potato ecotype-derived culture media (Control, *Condor Imilla*, *Yana Runa*, *Leke Pek’e*, *Luk’i Turno*, and *Dutch Désirée*) on the antifungal activity of *T. harzianum* BOL-12QD (ThB12QD) culture filtrates against *B. cinerea* (Bc10^6^; 10^6^ conidia mL^−1^). (**A**) Radial growth response of *B. cinerea* exposed to culture filtrates produced using different potato ecotypes. (**B**) *B. cinerea* growth expressed as a percentage of the untreated control (control = 100%). Percentage inhibition values reported in the text were calculated as 100 − % relative growth. Values are expressed as mean ± SD (n = 5 biological replicates). Different letters indicate statistically significant differences among treatments (one-way ANOVA followed by Tukey’s HSD, *p* < 0.05).

The influence of different potato ecotypes on the antifungal activity of *T. harzianum* BOL-12QD culture filtrates was evaluated based on their effects on *B. cinerea* growth. As illustrated in Figure 3B, fungal growth is expressed as a percentage of the untreated control (control = 100%), while antifungal activity is reported as percentage inhibition, calculated as 100 − % relative growth.

Statistical analysis indicated that potato ecotype significantly influenced the antifungal activity of the resulting culture filtrates (one-way ANOVA: F_5,12_ = 25.83; *p* < 0.0001).

Among the evaluated ecotypes, *Leke Pek’e* produced the lowest *B. cinerea* growth (48%; 52% inhibition), accompanied by a reduction in radial growth rate (4.1 mm d^−1^ vs. 5.6 mm d^−1^ in the standard medium) and a decrease in final colony diameter (16 mm vs. 33 mm), indicating the strongest inhibitory effect.

*Condor Imilla* and *Yana Runa* exhibited similar responses, with approximately 53% relative growth (47% inhibition), whereas *Luk’i Turno* displayed 50% relative growth (50% inhibition). These conditions formed an intermediate activity group with partial overlap in post hoc comparisons.

In contrast, *Dutch Désirée* resulted in higher relative growth (65%; 35% inhibition), indicating a weaker antifungal effect. The medium control exhibited 67% relative growth (33% inhibition), representing the lowest inhibition level observed among treatments.

Tukey’s HSD post hoc analysis confirmed *Leke Pek’e* as the highest-inhibitory treatment; however, partial overlaps were observed among *Condor Imilla*, *Yana Runa*, and *Luk’i Turno*, indicating modest differences among these ecotypes.

Overall, potato ecotype significantly influenced the antifungal activity of *T. harzianum* BOL-12QD culture filtrates, with *Leke Pek’e* consistently producing the strongest reduction in *B. cinerea* growth across evaluated parameters.

##### Nitrogen Source Composition

**Figure 4:**
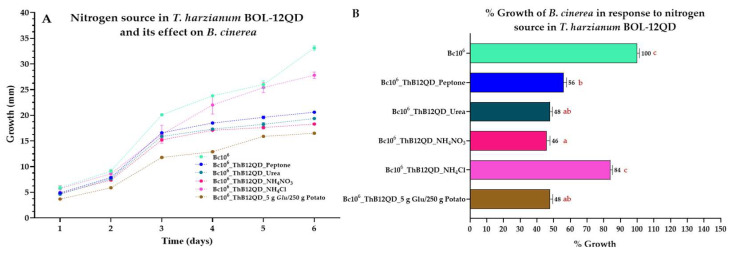
Effect of nitrogen source variation on the antifungal activity of *T. harzianum* BOL-12QD (ThB12QD) culture filtrates against *B. cinerea* (Bc10^6^; 10^6^ conidia mL^−1^). (**A**) Growth response of *B. cinerea* exposed to culture filtrates obtained under different nitrogen sources (peptone, urea, ammonium nitrate, ammonium chloride, and nitrogen-free control). (**B**) *B. cinerea* growth expressed as a percentage of the untreated control (control = 100%). Percentage inhibition values reported in the text were calculated as 100 − % relative growth. Values are expressed as mean ± SD (n = 5 biological replicates). Different letters indicate statistically significant differences among treatments (one-way ANOVA followed by Tukey’s HSD, *p* < 0.05).

The influence of nitrogen source composition on the antifungal activity of *T. harzianum* BOL-12QD culture filtrates against *B. cinerea* was evaluated using a basal medium in which the nitrogen source was systematically varied. As illustrated in Figure 4B, fungal growth is expressed as a percentage of the untreated control (control = 100%), while antifungal activity is reported as percentage inhibition, calculated as 100 − % relative growth.

Statistical analysis revealed a significant effect of nitrogen source on antifungal activity (one-way ANOVA: F_4,10_ = 28.67; *p* < 0.0001).

Ammonium nitrate and urea produced the lowest *B. cinerea* growth values (46% and 48%, corresponding to 54% and 52% inhibition, respectively). These treatments were not significantly different from each other (Tukey’s HSD, *p* = 0.078), indicating comparable antifungal performance.

Peptone resulted in intermediate activity (56% growth; 44% inhibition), whereas ammonium chloride exhibited the weakest effect (84% growth; 16% inhibition) and differed significantly from all other nitrogen sources (*p* < 0.001).

Consistent with these results, ammonium nitrate and urea were associated with reduced colony extension (15–16 mm), whereas peptone and ammonium chloride exhibited progressively higher radial growth.

Although inhibition values observed during nitrogen optimisation were numerically similar to those obtained in previous optimisation steps, these datasets were generated independently and are therefore not directly comparable within a unified quantitative framework.

Overall, nitrogen source significantly influenced the antifungal activity of *T. harzianum* BOL-12QD culture filtrates, with ammonium nitrate and urea producing the highest inhibition levels under the tested conditions, followed by peptone, while ammonium chloride consistently exhibited minimal activity.

#### 3.1.3. Effect of Biological Interactions

##### Co-Cultivation with *B. cinerea*

**Figure 5:**
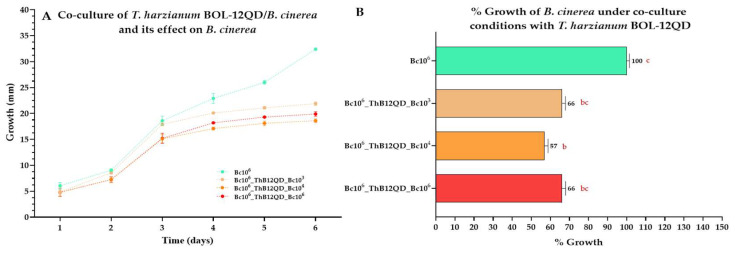
Effect of *B. cinerea* inoculum density during co-cultivation on the antifungal activity of *T. harzianum* BOL-12QD (ThB12QD) culture filtrates. (**A**) Radial growth rate of *B. cinerea* following exposure to culture filtrates obtained from co-cultures established with different inoculum densities (10^3^, 10^4^, and 10^6^ conidia mL^−1^). (**B**) *B. cinerea* growth expressed as a percentage of the untreated control (control = 100%). Percentage inhibition values reported in the text were calculated as 100 − % relative growth. Values are expressed as mean ± SD (n = 5 biological replicates). Different letters indicate statistically significant differences among treatments (one-way ANOVA followed by Tukey’s HSD, *p* < 0.05).

The effect of co-cultivation of *T. harzianum* BOL-12QD with *B. cinerea* on antifungal activity was evaluated by varying pathogen inoculum density (10^3^, 10^4^, and 10^6^ conidia mL^−1^) as a biological stimulus. As illustrated in Figure 5B, fungal growth is expressed as a percentage of the untreated control (control = 100%), while antifungal activity is reported as percentage inhibition, calculated as 100 − % relative growth.

Statistical analysis revealed a significant effect of co-cultivation conditions on fungal growth dynamics and antifungal activity (one-way ANOVA: F_3,8_ = 21.34; *p* < 0.0001).

At 10^3^ conidia mL^−1^, growth was reduced to approximately 66% of the control (34% inhibition; *p* < 0.05), accompanied by a growth rate of 5.51 d^−1^ and a maximum hyphal extension of 20 mm (*p* < 0.01 vs. control). At 10^4^ conidia mL^−1^, a stronger effect was observed, with growth reduced to 57% (43% inhibition; *p* < 0.01), a growth rate of 4.45 d^−1^ (*p* < 0.001), and a maximum hyphal extension of 17 mm (*p* < 0.001), representing the highest inhibitory response. In contrast, at 10^6^ conidia mL^−1^, growth reached 66% (34% inhibition; *p* < 0.05), with a growth rate of 4.80 d^−1^ and a maximum hyphal extension of 18 mm (*p* < 0.01).

The untreated control exhibited a growth rate of 5.41 d^−1^ and a maximum hyphal extension of 32 mm, with no detectable inhibition.

Tukey’s HSD post hoc analysis indicated a non-linear response to inoculum density. The 10^4^ conidia mL^−1^ condition exhibited significantly higher inhibitory activity than both the 10^3^ and 10^6^ treatments (*p* < 0.05), whereas no significant differences were detected between 10^3^ and 10^6^ conidia mL^−1^ (*p* > 0.05).

Overall, co-cultivation influenced the antifungal activity of *T. harzianum* BOL-12QD in a non-linear manner dependent on pathogen density, with 10^4^ conidia mL^−1^ acting as the most effective elicitor condition under the experimental setup.

##### Self-Inhibitory Activity of *B. cinerea* Culture Filtrates

**Figure 6:**
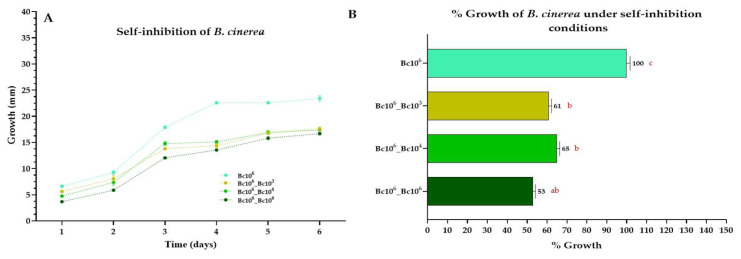
Self-inhibitory activity of *B. cinerea* culture filtrates obtained from cultures initiated with different inoculum densities (10^3^, 10^4^, and 10^6^ conidia mL^−1^). (**A**) Radial growth rate of *B. cinerea* exposed to self-derived culture filtrates. (**B**) *B. cinerea* growth expressed as a percentage of the untreated control (control = 100%). Percentage inhibition values reported in the text were calculated as 100 − % relative growth. Values are expressed as mean ± SD (n = 5 biological replicates). Different letters indicate statistically significant differences among treatments (one-way ANOVA followed by Tukey’s HSD, *p* < 0.05).

The autoinhibitory activity of *B. cinerea* culture filtrates was evaluated by assessing their effects on the growth of the same fungal species using filtrates generated from cultures initiated at different inoculum densities (10^3^, 10^4^, and 10^6^ conidia mL^−1^). As illustrated in Figure 6B, fungal growth is expressed as a percentage of the untreated control (control = 100%), while antifungal activity is reported as percentage inhibition, calculated as 100 − % relative growth.

Statistical analysis revealed a significant effect of filtrate origin on fungal growth dynamics (one-way ANOVA: F_3,8_ = 19.87; *p* < 0.0001).

Filtrates derived from 10^6^ conidia mL^−1^ cultures exhibited the strongest autoinhibitory effect, reducing *B. cinerea* growth to approximately 53% of the control (47% inhibition; *p* < 0.001) and limiting maximum hyphal extension to 12 mm. Filtrates from 10^3^ conidia mL^−1^ produced an intermediate response, with 61% relative growth (39% inhibition) and 14 mm hyphal extension (*p* < 0.001). In contrast, filtrates from 10^4^ conidia mL^−1^ exhibited the weakest inhibitory effect, with 65% relative growth (35% inhibition) and 15 mm hyphal extension.

The untreated control exhibited a radial growth rate of 5.65 d^−1^ and a maximum hyphal extension of 23 mm, with no detectable inhibition.

Tukey’s HSD post hoc analysis indicated a non-linear response, with the following overall ranking: 10^6^ conidia mL^−1^ > 10^3^ conidia mL^−1^ > 10^4^ conidia mL^−1^.

Overall, *B. cinerea* culture filtrates exhibited autoinhibitory activity dependent on the inoculum density used during culture establishment, indicating density-dependent regulation of extracellular metabolite production rather than a strictly proportional response.

##### Effect of Volatile Organic Compounds on *B. cinerea* Growth

**Figure 7:**
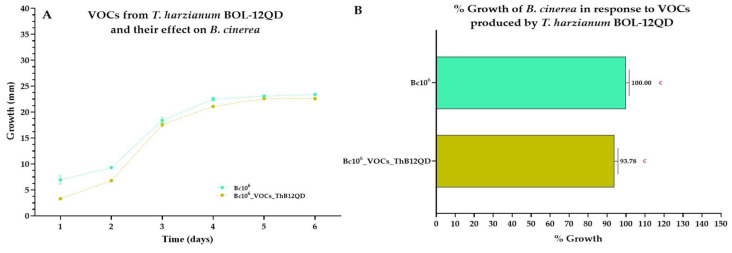
Effect of volatile organic compounds (VOCs) produced by *T. harzianum* BOL-12QD (ThB12QD) on the growth of *B. cinerea* (Bc10^6^; 10^6^ conidia mL^−1^). (**A**) Radial growth rate of *B. cinerea* under VOC exposure in a shared-headspace system. (**B**) *B. cinerea* growth expressed as a percentage of the untreated control (control = 100%). Percentage inhibition values reported in the text were calculated as 100 − % relative growth. Values are expressed as mean ± SD (n = 5 biological replicates). Different letters indicate statistically significant differences among treatments (one-way ANOVA followed by Tukey’s HSD, *p* < 0.05).

The influence of volatile organic compounds (VOCs) produced by *T. harzianum* BOL-12QD on the growth of *B. cinerea* was evaluated using a shared-headspace system that prevented any physical contact between the two fungi. Figure 7B illustrates that fungal growth is expressed as a percentage of the untreated control (control = 100%), while antifungal activity is reported as percentage inhibition, calculated as 100 − % relative growth.

Statistical analysis revealed no significant effect of VOC exposure on fungal growth under the conditions tested (one-way ANOVA: F_1,6_ = 2.14; *p* = 0.198).

Exposure to VOCs from *T. harzianum* resulted in a relative growth of 93.78% (6.22% inhibition), indicating only a marginal reduction compared with the control. No statistically significant differences were detected between treatments, and values remained within the range of experimental variability.

Accordingly, VOC exposure did not affect radial growth rate or colony development under the experimental conditions evaluated.

Overall, these findings indicate that VOCs produced by *T. harzianum* BOL-12QD did not exert a detectable antifungal effect in this system. In contrast with the strong activity observed in culture filtrate and co-culture assays, the results suggest that the antagonistic activity of this strain under the tested conditions is primarily associated with non-volatile, diffusible metabolites.

#### 3.1.4. Antifungal Activity Under Sequentially Optimised Conditions

**Figure 8:**
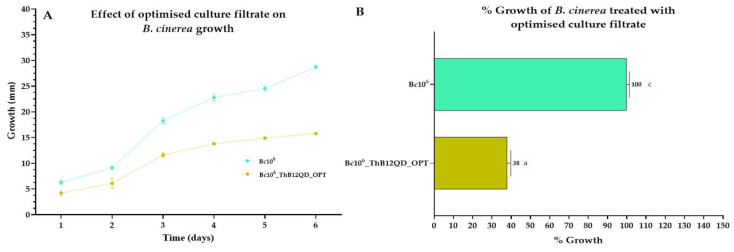
Antifungal activity of culture filtrates produced under sequentially optimised culture conditions against *B. cinerea*. (**A**) Growth kinetics of *B. cinerea* (Bc10^6^; 10^6^ conidia mL^−1^) exposed to the optimised *T. harzianum* BOL-12QD (Bc10^6^_ThB12QD_OPT) culture filtrate compared with the untreated control. (**B**) *B. cinerea* growth expressed as a percentage of the untreated control (control = 100%). Percentage inhibition values reported in the text were calculated as 100 − % relative growth. Values are expressed as mean ± SD (n = 5 biological replicates). Different letters indicate statistically significant differences among treatments (one-way ANOVA followed by Tukey’s HSD, *p* < 0.05).

The antifungal activity of *T. harzianum* BOL-12QD culture filtrates was further evaluated using a sequential optimisation approach, in which the best-performing condition from each independent experimental category (physical, chemical, and biological parameters) was selected. As previously described, fungal growth in Figure 8B is expressed as a percentage relative to the untreated control (control = 100%), and antifungal activity is reported as percentage inhibition calculated as 100 − % relative growth.

This sequential strategy is based on independent single-factor optimisation experiments and does not constitute a factorial design; therefore, interaction effects between variables cannot be inferred.

From the physical parameter set, the violet light-filter condition was selected due to its highest antifungal activity (47% relative growth; 53% inhibition). For carbon source optimisation, the combination of 5 g L^−1^ glucose and 250 g L^−1^ potato extract (*Leke Pek’e* ecotype) was selected based on consistent performance (48% inhibition; 52% relative growth). For nitrogen source optimisation, ammonium nitrate was selected as the most effective condition (46% relative growth; 54% inhibition). In the biological interaction category, co-cultivation with *B. cinerea* at 10^4^ conidia mL^−1^ was selected due to its highest inhibitory effect (43% inhibition).

VOCs were not included in the optimisation framework due to the absence of a statistically significant effect on fungal growth (*p* = 0.198), whereas self-inhibitory activity of *B. cinerea* was excluded as it represents an intrinsic physiological property of the pathogen rather than a modifiable parameter of the production system.

The selected conditions were integrated into a single production protocol, and cultures were incubated at 25 °C under violet-filter illumination using the optimised nutritional and biological conditions described above.

The antifungal activity of the combined filtrate was evaluated against *B. cinerea* over a 6-day period (Figure 8A). In the untreated control, radial growth increased from 6.28 mm on day 1 to 28.74 mm on day 6. In treated cultures, growth ranged from 4.20 mm to 15.80 mm over the same period.

Statistical analysis confirmed significant differences between treatments (one-way ANOVA: F_1,10_ = 24.87; *p* < 0.001). At day 6, treated colonies reached 55% of the radial growth observed in the control, corresponding to 45% inhibition. This endpoint effect was consistent with the overall inhibition value (62%) derived from Figure 8B, reflecting differences between kinetic and endpoint-based calculations.

Overall, the sequential combination of independently optimised parameters resulted in improved antifungal performance compared with single-factor conditions. However, in the absence of a factorial design, these results should be interpreted as additive effects of independently optimised variables rather than synergistic or interaction-driven responses.

The optimised culture conditions were subsequently used for metabolomic fingerprinting and biological safety assessments described in the following sections.

### 3.2. Comparative ^1^H NMR Metabolic Fingerprinting of Culture Filtrates

**Figure 9:**
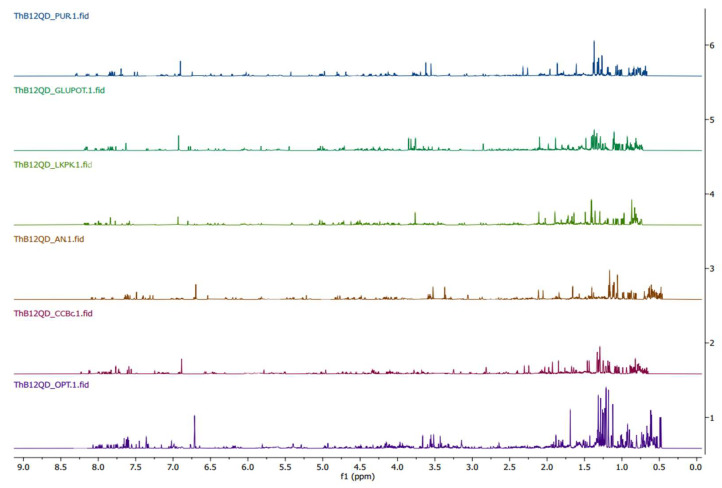
^1^H NMR spectra (500 MHz, CD_3_OD) of culture filtrates obtained from *T. harzianum* BOL-12QD grown under different optimisation conditions: ThB12QD_PUR (violet colour-filter condition); ThB12QD_GLUPOT (5 g L^−1^ glucose and 250 g L^−1^ potato extract); ThB12QD_LKPK (Leke Pek’e potato ecotype); ThB12QD_AN (ammonium nitrate supplementation); ThB12QD_CCBc (co-culture with *B. cinerea*, 10^4^ conidia mL^−1^); and ThB12QD_OPT (combined optimised condition).

Aliquots (100 mL) of culture filtrates were collected from *T. harzianum* BOL-12QD cultures grown under the selected physical, chemical, and biological conditions identified during the optimisation process, including violet-filter cultivation, medium supplemented with 5 g L^−1^ glucose and 250 g L^−1^ potato extract, medium prepared using the *Leke Pek’e* potato ecotype, ammonium nitrate supplementation, co-cultivation with *B. cinerea* (10^4^ conidia mL^−1^), and the final optimised integrated condition. All filtrates were lyophilised, reconstituted in CD_3_OD, and analysed by ^1^H NMR spectroscopy (500 MHz) under identical acquisition parameters (Figure 9).

The resulting spectra exhibited qualitative differences in signal distribution and spectral complexity among culture conditions, reflecting variation in extracellular metabolite fingerprints. As all samples were processed under identical extraction, lyophilisation, and acquisition conditions, these differences are attributable to variations in the chemical composition of the corresponding culture filtrates.

All conditions exhibited resonances across the main spectral regions, including aromatic (*δ_H_* 8.32–6.80), olefinic (*δ_H_* 6.60–5.18), oxygenated (*δ_H_* 5.08–3.00), and aliphatic (*δ_H_* 3.13–0.44) regions, although differences in signal density and overlap were observed between treatments.

In particular, the filtrate obtained under violet-filter conditions and that produced using 5 g L^−1^ glucose combined with 250 g L^−1^ potato extract exhibited increased signal density in the oxygenated region (approximately *δ_H_* 5.0–3.0), suggesting differences in metabolite distribution within this chemical shift range. Similar overall spectral patterns were observed for the *Leke Pek’e* ecotype and ammonium nitrate conditions, with minor variations in signal intensity distribution across oxygenated and aliphatic regions.

The co-culture condition with *B. cinerea* (10^4^ conidia mL^−1^) exhibited broader signal dispersion and increased overlap, particularly within the oxygenated and aliphatic regions, compared with several monoculture-derived filtrates.

Among all conditions, the optimised culture filtrate (ThB12QD_OPT) exhibited the most complex spectral profile, with resonances spanning the full spectral window (*δ_H_* 8.32–0.44) and increased signal density across aromatic, olefinic, oxygenated, and aliphatic regions, indicative of broader extracellular chemical diversity relative to individual optimisation conditions.

Although differences in spectral fingerprints were observed among treatments, this analysis is restricted to comparative ^1^H NMR profiling of crude extracts. Accordingly, individual metabolite identification remains tentative, and definitive structural assignment would require complementary analytical approaches such as LC–MS/MS, high-resolution mass spectrometry, two-dimensional NMR experiments, and comparison with authentic standards.

### 3.3. Biological Safety Evaluation of the Optimised Culture Filtrate

#### 3.3.1. In Vitro Cytotoxicity Assessment in Murine Peritoneal Macrophages

The cytotoxic potential of the optimised *T. harzianum* BOL-12QD culture filtrate was evaluated using the MTT assay in murine peritoneal macrophages. Cell viability was estimated based on mitochondrial metabolic activity and expressed relative to untreated control cells.

The untreated control exhibited a mean absorbance of 2.38 and was defined as 100% viability. Exposure to the culture filtrate at concentrations ranging from 25 to 200 μg mL^−1^ did not reduce cellular metabolic activity compared with the control group (Table 1).

Across all tested concentrations, absorbance values ranged from 2.63 to 2.81, corresponding to relative viabilities between 110.5% and 118.1%. No concentration-dependent decrease in viability was observed, and all values remained at or above control levels.

Statistical analysis confirmed no significant differences among treatment groups (one-way ANOVA: F_3,12_ = 1.67; *p* = 0.234), and Tukey’s post hoc test did not identify any significant pairwise differences (*p* > 0.05).

Overall, the optimised *T. harzianum* BOL-12QD culture filtrate did not induce detectable cytotoxic effects in murine peritoneal macrophages under the experimental conditions evaluated.

#### 3.3.2. Oral Toxicity Outcomes In Vivo

The potential toxicity of the optimised *T. harzianum* BOL-12QD culture filtrate was evaluated in mice following repeated oral administration at 250 mg kg^−1^ d^−1^ for 10 consecutive days. Animals were monitored daily for predefined clinical observations.

No positive observations were recorded in control animals throughout the study period (0/5 animals) (Table 2).

In treated animals, positive observations appeared progressively over time. The first occurrence was detected on day 5 in one animal (1/5; 20%). The number of affected animals increased to 3/5 (60%) on day 6 and 4/5 (80%) on day 7. From day 8 onwards, all treated animals exhibited positive observations (5/5; 100%), which remained constant until the end of the study (days 9–10).

The mean latency to first observation was 6.4 days, indicating a delayed onset of treatment-associated effects during repeated exposure.

Trend analysis revealed a significant increase in the incidence of positive observations over time (Cochran–Armitage trend test, *p* < 0.0001). Final incidence differed significantly between treated and control groups (Fisher’s exact test, *p* < 0.0001).

No mortality was observed in either group during the experimental period.

Overall, repeated oral administration of the optimised *T. harzianum* BOL-12QD culture filtrate resulted in a time-dependent increase in the frequency of clinical observations under the conditions tested.

## 4. Discussion

The results obtained in this study demonstrate that the inhibitory activity of *T. harzianum* BOL-12QD against *B. cinerea* arises from a complex interplay of physical, chemical and biological factors that collectively regulate fungal physiological performance. Systematic optimisation of these variables significantly enhanced antifungal activity, reaching inhibition values of up to 54% under individual optimal conditions and 62% under fully integrated conditions. Although the present experimental design does not allow formal statistical evaluation of interaction effects, the observed increase suggests a functional convergence of multiple regulatory inputs, potentially reflecting coordinated metabolic reprogramming under combined environmental and nutritional cues. These findings are consistent with high-performance biocontrol strains of *Trichoderma* and support the biotechnological potential of this isolate.

First, it should be explicitly clarified that whole-genome sequencing (WGS) and comparative genomic analyses were not performed for the BOL-12QD isolate. Consequently, no assumption is made regarding genomic identity, functional equivalence, or conservation of complete metabolic networks between this strain and other *T. harzianum* isolates described in the literature. The molecular characterisation available for BOL-12QD is limited to the ribosomal ITS1–5.8S–ITS2 region, obtained by Sanger sequencing and deposited under GenBank accession KY644517.1. While this marker provides robust taxonomic identification within *Trichoderma harzianum* (*Hypocreales*: *Hypocreaceae*), it is highly conserved and does not permit inference of genome-wide similarity, biosynthetic gene cluster conservation, or equivalence of complex regulatory systems [38].

To contextualise intraspecific variability, the BOL-12QD isolate can be compared with *T. harzianum* strain T2 (GenBank accession OP010005.1), which provides a useful reference point for illustrating diversity within the species. Although ITS-based analysis confirms species-level identity, these isolates exhibit differences in ITS sequence length (576 bp for T2 versus 524 bp for BOL-12QD) and originate from markedly distinct ecological niches, with strain T2 recovered from blue-stained *Pinus thunbergii* wood in China [39], whereas BOL-12QD was isolated in Bolivia. Taken together, these observations underscore the substantial ecological and phylogenetic heterogeneity that exists within *T. harzianum*, even among isolates classified within the same species complex.

Importantly, the mechanistic interpretations presented in this study are not based on assumptions of genomic identity between strains. Instead, they rely on evidence supporting functional conservation of key signalling and antagonistic systems within the genus *Trichoderma*. Transcriptomic analyses in *T. harzianum* T2 following induction with Botrytis cinerea cell wall components have demonstrated coordinated activation of genes involved in mycoparasitism, including G protein-mediated signalling pathways, MAPK cascades, carbohydrate-active enzymes (CAZymes), major facilitator superfamily (MFS) transporters, and oxidative stress response systems. These conserved modules provide a mechanistic framework for cautious extrapolation of regulatory behaviour in BOL-12QD, without implying genomic equivalence [40].

Among the physical parameters evaluated, light quality exerted a measurable effect on antifungal performance. The violet-filter condition produced the highest inhibition (53%), suggesting that spectral composition acts as an environmental cue influencing fungal physiology. Light-mediated regulation of development and secondary metabolism has been widely reported in *Trichoderma* species, where photoreceptor systems such as *BLR1/BLR2* coordinate development, sporulation and metabolic output in *T. atroviride*, whilst *ENVOY*- and *MAPK*-associated pathways contribute to environmental adaptation in *T. guizhouense* [41,42,43,44,45,46,47]. These observations suggest that BOL-12QD may respond to high-energy light stress through conserved photoregulatory networks, potentially modulating antagonistic capacity. However, in the absence of irradiance and spectral characterisation, these effects should be interpreted as responses to operational light-filter conditions rather than wavelength-specific mechanisms.

Light also influences fungal morphology, colony architecture and antagonistic performance in several *Trichoderma* species. In *T. guizhouense*, red and far-red light enhance hyphal organisation and pathogen interaction efficiency [47]. Such responses may indirectly contribute to antifungal activity in BOL-12QD through modulation of metabolic allocation and secondary metabolite production. These processes are likely integrated via conserved signalling pathways, including *BLR1/BLR2*, *ENVOY*, *cAMP/PKA* and *MAPK* cascades, which connect environmental perception with metabolic regulation [48,49,50].

Culture medium composition also strongly influenced antifungal activity. Intermediate glucose concentration (5 g L^−1^) combined with high potato extract (250 g L^−1^) produced the highest inhibition, whereas elevated glucose levels reduced activity. This pattern is consistent with carbon catabolite repression mediated by *Cre1*, which suppresses secondary metabolism under high glucose availability [51]. Under moderate carbon limitation, derepression of global regulators such as *Lae1* and *Xpp1* may facilitate the transition from trophophase to idiophase, enhancing production of hydrolytic enzymes and secondary metabolites [51].

Potato extracts likely function as a complex nutritional matrix containing starch-derived oligosaccharides and micronutrients that influence fungal physiology and metabolic flux distribution, potentially affecting *NRPS* and *PKS* pathways [52]. Similar substrate-dependent regulation of secondary metabolism has been reported in *Trichoderma*-based systems, where carbon heterogeneity modulates enzyme secretion and antifungal efficiency under nutrient-limited conditions [52,53,54].

Variability among potato ecotypes also influenced antifungal activity. *Luk’i Turno* produced the highest inhibition, followed by *Condor Imilla* and *Yana Runa*, whereas the *Dutch Désirée* control exhibited the lowest activity. These differences may reflect variation in starch polysaccharide composition and associated biomolecules, which could influence carbon availability and metabolic activation in *T. harzianum*.

The potato ecotypes evaluated differ in starch composition, particularly in amylose and amylopectin ratios, which constitute the main carbohydrate fraction of tubers. Although no independent compositional analysis was performed, characterisation reported by Valdivieso [55] indicates that *Luk’i Turno* and *Dutch Désirée* contain approximately 25% amylose and 75% amylopectin, *Yana Runa* presents an intermediate composition of approximately 30% amylose, whereas *Condor Imilla* exhibits a higher amylose content (37.12%). *Leke Pek’e*, which showed the strongest antifungal activity, also presents an approximately 25/75 amylose-to-amylopectin ratio.

These compositional differences may influence starch hydrolysis kinetics, carbon release dynamics and the profile of carbohydrate-derived substrates available to *T. harzianum*. Such effects may in turn modulate fungal metabolism and indirectly affect secondary metabolite production. Accordingly, the superior performance observed with *Leke Pek’e* may be partially associated with its starch composition, although this relationship remains inferential. Nevertheless, other tuber constituents such as proteins, micronutrients and phenolic compounds likely contribute to the observed responses, indicating a multifactorial basis.

Nitrogen availability further modulated antifungal activity. Ammonium nitrate yielded the highest inhibition, followed by urea and ammonium chloride. This effect may result from nitrogen-dependent regulation of fungal metabolism, including intracellular pH modulation, carbon–nitrogen balance and activation of lytic enzyme systems such as chitinases and *β*-1,3-glucanases [56]. Comparable nitrogen-dependent modulation of antagonistic activity has been reported in *Trichoderma* species [56,57,58].

Biotic interaction with *B. cinerea* further enhanced antifungal activity. Co-cultivation at 10^4^ conidia mL^−1^ induced the highest inhibition (43%), suggesting an optimal elicitation threshold. This dose-dependent response may reflect a quorum-sensing-like mechanism, whereby pathogen-derived signals activate *MAPK* and *cAMP/PKA* signalling cascades, leading to the induction of mycoparasitism-related genes such as *chit42* and *prb1* [59,60,61]. Similar inducible systems have been reported in *Trichoderma*, where chitinases, glucanases and proteases play central roles in host cell wall degradation and antagonistic activity. At higher pathogen densities (10^6^ conidia mL^−1^), inhibition decreased to 34%, which may indicate saturation or feedback regulation of recognition and signalling pathways.

Conversely, *B. cinerea* itself exhibited density-dependent self-inhibitory behaviour at higher conidial concentrations, reaching 47% inhibition at 10^6^ conidia mL^−1^. This response may be associated with the accumulation of endogenous secondary metabolites, such as botcinins and sesquiterpenes, which have been reported to interfere with cytoskeletal dynamics and fungal development [62]. Comparable density-dependent regulation has been observed in other filamentous fungi, including *Geotrichum candidum*, where volatile metabolites modulate germination and hyphal growth [63,64], suggesting that self-inhibition in *B. cinerea* may arise from conserved stress- and metabolite-mediated regulatory processes.

Volatile organic compounds produced by *T. harzianum* BOL-12QD showed only minimal inhibitory activity (6.2%), suggesting that VOC-mediated antagonism is not a major mechanism under the tested conditions. This contrasts with VOC-active *Trichoderma* strains reported in the literature, where inhibitory effects can reach substantially higher levels depending on environmental context [65,66]. The slight stimulation of pathogen growth observed may indicate metabolic adaptation or CO_2_-mediated enhancement of *B. cinerea* growth, further supporting the limited contribution of volatile-mediated interactions in this system and reinforcing the predominance of contact-dependent and diffusible metabolite-driven mechanisms in this strain.

When all optimised variables were integrated, antifungal activity increased to 62%. Although interaction effects cannot be statistically resolved, this outcome suggests functional convergence of multiple regulatory inputs under combined environmental and nutritional conditions. Light signalling, carbon regulation, nitrogen assimilation and biotic stress responses likely act in concert to enhance metabolic output.

At the metabolic level, this integrated state is associated with a chemically diverse exometabolome comprising peptaibols, polyketides, terpenoids and hydrolytic enzymes. These metabolites may act synergistically through membrane disruption, enzymatic degradation of cell walls and interference with fungal signalling pathways. Although direct metabolite–function relationships were not established, this profile is consistent with previous metabolomic studies in *T. harzianum* [67,68,69,70,71,72].

^1^H NMR profiling confirms this metabolic integration, revealing simultaneous expression of aromatic, aliphatic and oxygenated metabolites consistent with polyketide, peptide and terpenoid biosynthesis [67,68,69,70,71,72]. Unlike earlier studies reporting these metabolites in isolation, the present results indicate their concurrent activation under multifactorial conditions, supporting a more integrated biosynthetic framework in *Trichoderma*.

This metabolic plasticity aligns with the broad chemical diversity reported for *T. harzianum*, which produces over 180–200 secondary metabolites [72]. The present findings extend this view by suggesting that environmental integration may trigger simultaneous activation of multiple biosynthetic pathways, generating emergent chemical profiles not predictable from single-factor studies.

Finally, cytotoxicity assays confirmed that *T. harzianum* BOL-12QD culture filtrates are non-toxic to mammalian cells across the tested range (25–200 µg mL^−1^), consistent with previous reports of low cytotoxicity in *Trichoderma*-derived extracts [73,74]. In vivo evaluations indicated high tolerability at elevated doses, although reversible physiological effects were observed. Overall, these results support a favourable safety margin consistent with previous toxicological studies of *Trichoderma* species [75,76].

The main limitation of this study is the absence of a factorial experimental design and targeted metabolomic validation, which restricts mechanistic inference regarding specific bioactive compounds. It is further emphasised that no compositional analysis of potato ecotypes was performed, and all substrate-related interpretations are based on external literature [55]. Similarly, no genomic data beyond ITS-based identification are available for strain BOL-12QD; therefore, all mechanistic interpretations must be considered functional extrapolations grounded in conserved genus-level biology rather than strain-specific genomic evidence. Future studies incorporating controlled interaction designs and high-resolution metabolomics (LC–MS/MS and 2D NMR) will be essential to link environmental modulation with specific bioactive metabolites and genetic determinants.

## 5. Conclusions

Optimisation of physical, chemical and biological culture parameters was associated with a progressive increase in the antifungal activity of *T. harzianum* BOL-12QD culture filtrates against *B. cinerea*, reaching inhibition levels of up to 62% under fully integrated conditions. The most effective production system comprised violet-filter illumination, a medium supplemented with 5 g L^−1^ glucose and 250 g L^−1^ potato extract, the *Leke Pek’e* potato ecotype, ammonium nitrate as the nitrogen source, and co-cultivation with *B. cinerea* at 10^4^ conidia mL^−1^.

These results indicate that antifungal performance is strongly influenced by the combined effects of environmental, nutritional and biological culture parameters. However, as interaction effects were not assessed within a factorial experimental design, the observed improvement should be interpreted as the cumulative outcome of sequential optimisation rather than evidence of synergistic interactions.

^1^H NMR fingerprinting revealed condition-dependent modulation of extracellular metabolite profiles, with the optimised condition exhibiting the most distinct spectral signature. This suggests that multifactorial culture environments influence the overall complexity and organisation of the exometabolome. Nevertheless, definitive structural assignment and pathway-level interpretation will require complementary approaches, including LC–MS/MS and high-resolution NMR-based structural elucidation.

The optimised culture filtrate showed no cytotoxic effects in murine peritoneal macrophages at concentrations up to 200 µg mL^−1^. However, repeated oral administration in mice induced observable treatment-related clinical signs in the absence of mortality, indicating that further toxicological characterisation is required to clarify the biological relevance of prolonged exposure and potential in vivo metabolite transformation.

All mechanistic interpretations presented in this study are based on functional conservation within the genus *Trichoderma*, rather than strain-specific genomic evidence, as no whole-genome sequencing data were generated for strain BOL-12QD.

Overall, *T. harzianum* BOL-12QD represents a promising source of bioactive metabolites with potential applications in biocontrol strategies. Future work should focus on targeted metabolite identification, mechanistic validation of antifungal pathways, expanded toxicological assessment, and evaluation of efficacy under greenhouse and field conditions.

## Figures and Tables

**Table 1:** Cytotoxicity assessment of the optimised *T. harzianum* BOL-12QD culture filtrate in murine peritoneal macrophages after 3 h of exposure, determined using the MTT assay. Results are expressed as mean absorbance values and relative cell viability (%) compared with untreated control cells.

Sample	Mean	% Cell Growth Inhibition	Toxicity
Control	2.38	–	–
SFOThB12QD (200 μg mL^−1^)	2.78	6.8%	No toxicological concern at tested concentration
SFOThB12QD (100 μg mL^−1^)	2.63	5.0%	Non-toxic
SFOThB12QD (50 μg mL^−1^)	2.68	3.2%	Non-toxic
SFOThB12QD (25 μg mL^−1^)	2.81	0.0%	Non-toxic

**Table 2:** Daily incidence of clinical observations recorded in mice receiving the optimised *T. harzianum* BOL-12QD culture filtrate (250 mg kg^−1^ d^−1^) or vehicle control during the 10-day exposure period. Positive (+) and negative (−) observations were recorded according to the predefined monitoring criteria.

Day	Control/Behaviour					SFOThB12QD/Behaviour				
	Mouse 1	Mouse 2	Mouse 3	Mouse 4	Mouse 5	Mouse 1	Mouse 2	Mouse 3	Mouse 4	Mouse 5
1	(−)	(−)	(−)	(−)	(−)	(−)	(−)	(−)	(−)	(−)
2	(−)	(−)	(−)	(−)	(−)	(−)	(−)	(−)	(−)	(−)
3	(−)	(−)	(−)	(−)	(−)	(−)	(−)	(−)	(−)	(−)
4	(−)	(−)	(−)	(−)	(−)	(−)	(−)	(−)	(−)	(−)
5	(−)	(−)	(−)	(−)	(−)	(−)	(−)	(−)	(+)	(−)
6	(−)	(−)	(−)	(−)	(−)	(+)	(−)	(−)	(+)	(+)
7	(−)	(−)	(−)	(−)	(−)	(+)	(+)	(−)	(+)	(+)
8	(−)	(−)	(−)	(−)	(−)	(+)	(+)	(+)	(+)	(+)
9	(−)	(−)	(−)	(−)	(−)	(+)	(+)	(+)	(+)	(+)
10	(−)	(−)	(−)	(−)	(−)	(+)	(+)	(+)	(+)	(+)

## Data Availability

The original contributions presented in this study are included in the article/Supplementary Material. Further inquiries can be directed to the corresponding author.

## Supplementary Materials

The following supporting information can be downloaded at: https://www.mdpi.com/article/10.3390/microorganisms14061331/s1, Figure S1. Growth of *Trichoderma harzianum* BOL-12QD in darkness; Figure S2. Growth of *Trichoderma harzianum* BOL-12QD under a blue light filter; Figure S3. Growth of *Trichoderma harzianum* BOL-12QD under a yellow light filter; Figure S4. Growth of *Trichoderma harzianum* BOL-12QD under a green light filter; Figure S5. Growth of *Trichoderma harzianum* BOL-12QD under a violet light filter; Figure S6. Growth of *Botrytis cinerea* under a violet light filter; Figure S7. Enzyme production by *Trichoderma harzianum* BOL-12QD under different light filters; Figure S8. Variability in mycelial growth and enzyme production by *Trichoderma harzianum* BOL-12QD under different light filters; Figure S9. 3^2^ factorial designs: variables—potato proportion and glucose concentration; Figure S10. Potato ecotypes used for enzyme production by *Trichoderma harzianum* BOL-12QD; Figure S11. Culture filtrates of *Trichoderma harzianum* BOL-12QD obtained using different potato ecotypes: *Condor Imilla* (*Solanum tuberosum* ssp. *andigena*), *Yana Runa* (*Solanum tuberosum* ssp. *andigena*), *Leke Pek’e* (*Solanum tuberosum* ssp. *andigena*), and *Luk’i Turno* (*Solanum* × *juzepczukii*); Figure S12. Co-culture: culture filtrates of *Trichoderma harzianum* BOL-12QD, *Botrytis cinerea*, and their co-culture; Figure S13. Culture filtrate of *Botrytis cinerea;* Figure S14. Inhibition of *Botrytis cinerea* growth by volatile organic compounds (VOCs) produced by *Trichoderma harzianum* BOL-12QD; Figure S15. ^1^H NMR spectrum (500 MHz, CD_3_OD) of the culture filtrate of *Trichoderma harzianum* BOL-12QD grown under violet light filter; Figure S16. ^1^H NMR spectrum (500 MHz, CD_3_OD) of the culture filtrate of *Trichoderma harzianum* BOL-12QD grown in a medium containing 5 g glucose and 250 g potato; Figure S17. ^1^H NMR spectrum (500 MHz, CD_3_OD) of the culture filtrate of *Trichoderma harzianum* BOL-12QD grown in a potato medium using the Leke Pek’e cultivar; Figure S18. ^1^H NMR spectrum (500 MHz, CD_3_OD) of the culture filtrate of *Trichoderma harzianum* BOL-12QD grown in a potato medium supplemented with ammonium nitrate as the nitrogen source; Figure S19. ^1^H NMR spectrum (500 MHz, CD_3_OD) of the culture filtrate of *Trichoderma harzianum* BOL-12QD in co-culture with *Botrytis cinerea* using a 10^4^ conidia mL^-1^ suspension; Figure S20. ^1^H NMR spectrum (500 MHz, CD_3_OD) of the optimised culture filtrate of *Trichoderma harzianum* BOL-12QD grown under violet light in a medium containing 5 g glucose and 250 g potato (Leke Pek’e cultivar), supplemented with ammonium nitrate, and in co-culture with *Botrytis cinerea* using a 10^4^ conidia mL^-1^ suspension.

## Abbreviations

The following abbreviations are used in this manuscript:

ABC	ATP-Binding Cassette Transporter
Aib	α-Aminoisobutyric Acid
AreA	Area Nitrogen Regulatory Transcription Factor
BLR	Blue Light Regulator
cAMP	Cyclic Adenosine Monophosphate
CHIT	Chitinase
Cre1	Carbon Catabolite Repressor 1
EGL1	Endoglucanase 1
ENVOY	PAS/LOV Photoreceptor Regulatory Protein
FAD	Flavin Adenine Dinucleotide
FMN	Flavin Mononucleotide
Gtt1	High-Affinity Glucose Transporter 1
HSD	Honestly Significant Difference
ISR	Induced Systemic Resistance
LOAEL	Lowest Observed Adverse Effect Level
LOV	Light, Oxygen or Voltage Photoreceptor Domain
NRPS	Non-Ribosomal Peptide Synthetase
PKS-NRPS	Hybrid Polyketide Synthase–Non-Ribosomal Peptide Synthetase System
PRR	Pattern Recognition Receptor
PVDF	Polyvinylidene Fluoride
VOCs	Volatile Organic Compounds
