# Supplementary material for: Optimisation of Culture Conditions Enhances Antifungal Activity and Reshapes Extracellular Metabolite Profiles in Trichoderma harzianum BOL-12QD
Source: Microorganisms. 2026 Jun 13;14(6):1331. doi: 10.3390/microorganisms14061331 (PMC13304288; doi:10.3390/microorganisms14061331)
Supplement: Supplementary file 1 [file microorganisms-14-01331-s001.zip › microorganisms-4342117-supplementary.pdf]

Supplementary Materials

Optimisation of Culture Conditions Enhances Antifungal Activity and Reshapes Extracellular Metabolite Profiles in *Trichoderma harzianum* BOL-12QD

Luis Apaza Ticona[1,*], María Teresa Alvarez Aliaga[2]

[1] Organic Chemistry Unit, Department of Chemistry in Pharmaceutical Sciences, Faculty of Pharmacy, University Complutense of Madrid, Plza. Ramón y Cajal s/n, 28040 Madrid, Spain
[2] Molecular Biochemistry Area, Instituto de Investigaciones Fármaco Bioquímicas, Universidad Mayor de San Andrés, Av. Saavedra No 2224, Zona Miraflores, La Paz 00067, Bolivia; mtalvarez@umsa.bo
* Correspondence: lnapaza@ucm.es

Contents:

- **Figure S1**. Growth of *Trichoderma harzianum* BOL-12QD in darkness.
- **Figure S2**. Growth of *Trichoderma harzianum* BOL-12QD under a blue light filter.
- **Figure S3**. Growth of *Trichoderma harzianum* BOL-12QD under a yellow light filter.
- **Figure S4**. Growth of *Trichoderma harzianum* BOL-12QD under a green light filter.
- **Figure S5**. Growth of *Trichoderma harzianum* BOL-12QD under a violet light filter.
- **Figure S6**. Growth of *Botrytis cinerea* under a violet light filter.
- **Figure S7**. Enzyme production by *Trichoderma harzianum* BOL-12QD under different light filters.
- **Figure S8**. Variability in mycelial growth and enzyme production by *Trichoderma harzianum* BOL-12QD under different light filters.
- **Figure S9**. 3^2 factorial designs: variables — potato proportion and glucose concentration.
- **Figure S10**. Potato ecotypes used for enzyme production by *Trichoderma harzianum* BOL-12QD.
- **Figure S11**. Culture filtrates of *Trichoderma harzianum* BOL-12QD obtained using different potato ecotypes: *Condor Imilla* (*Solanum tuberosum* ssp. *andigena*), *Yana Runa* (*Solanum tuberosum* ssp. *andigena*), *Leke Pek'e* (*Solanum tuberosum* ssp. *andigena*), and *Luk'i Turno* (*Solanum* × *juzepczukii*).
- **Figure S12**. Co-culture: culture filtrates of *Trichoderma harzianum* BOL-12QD, *Botrytis cinerea*, and their co-culture.
- **Figure S13**. Culture filtrate of *Botrytis cinerea*.
- **Figure S14**. Inhibition of *Botrytis cinerea* growth by volatile organic compounds (VOCs) produced by *Trichoderma harzianum* BOL-12QD.
- **Figure S15**. ^{1}H NMR spectrum (500 MHz, CD_3OD) of the culture filtrate of *Trichoderma harzianum* BOL-12QD grown under violet light filter.
- **Figure S16**. ^{1}H NMR spectrum (500 MHz, CD_3OD) of the culture filtrate of *Trichoderma harzianum* BOL-12QD grown in a medium containing 5 g glucose and 250 g potato.
- **Figure S17**. ^{1}H NMR spectrum (500 MHz, CD_3OD) of the culture filtrate of *Trichoderma harzianum* BOL-12QD grown in a potato medium using the Leke Pek'e cultivar.
- **Figure S18**. ^{1}H NMR spectrum (500 MHz, CD_3OD) of the culture filtrate of *Trichoderma harzianum* BOL-12QD grown in a potato medium supplemented with ammonium nitrate as the nitrogen source.
- **Figure S19**. ^{1}H NMR spectrum (500 MHz, CD_3OD) of the culture filtrate of *Trichoderma harzianum* BOL-12QD in co-culture with *Botrytis cinerea* using a 10^4 conidia mL^{-1} suspension.
- **Figure S20**. ^{1}H NMR spectrum (500 MHz, CD_3OD) of the optimised culture filtrate of *Trichoderma harzianum* BOL-12QD grown under violet light in a medium containing 5 g glucose and 250 g potato (Leke Pek'e cultivar), supplemented with ammonium nitrate, and in co-culture with *Botrytis cinerea* using a 10^4 conidia mL^{-1} suspension.

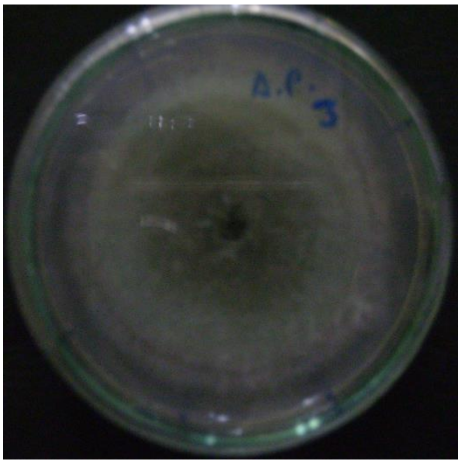

- **Figure S1**. Growth of *Trichoderma harzianum* BOL-12QD in darkness.

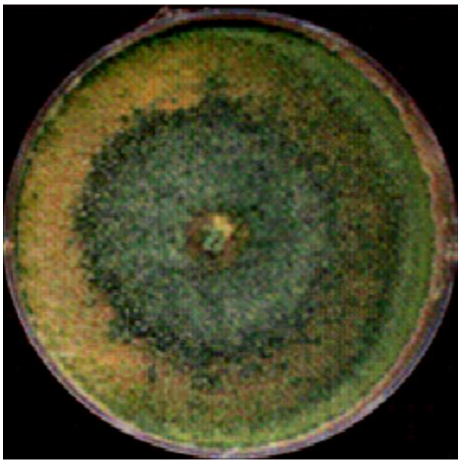

- **Figure S2**. Growth of *Trichoderma harzianum* BOL-12QD under a blue light filter.

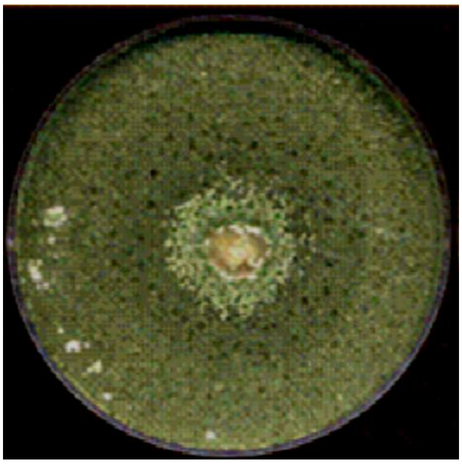

- **Figure S3**. Growth of *Trichoderma harzianum* BOL-12QD under a yellow light filter.

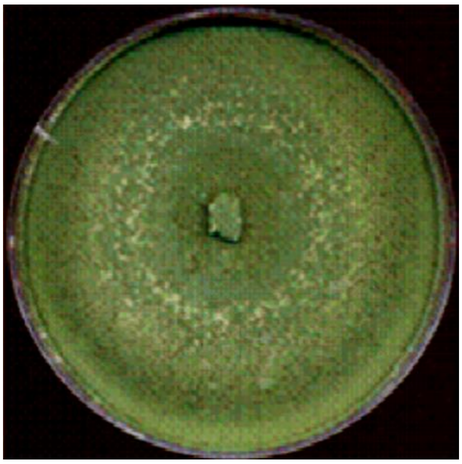

- **Figure S4**. Growth of *Trichoderma harzianum* BOL-12QD under a green light filter.

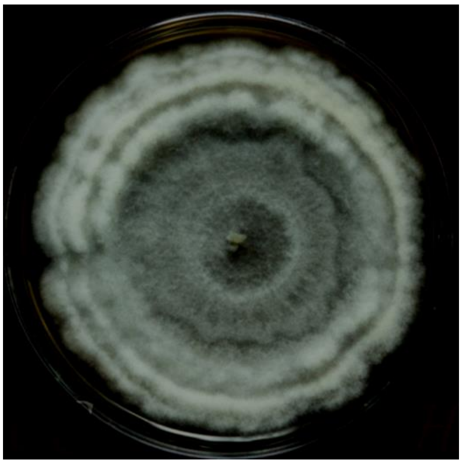

- **Figure S5**. Growth of *Trichoderma harzianum* BOL-12QD under a violet light filter.

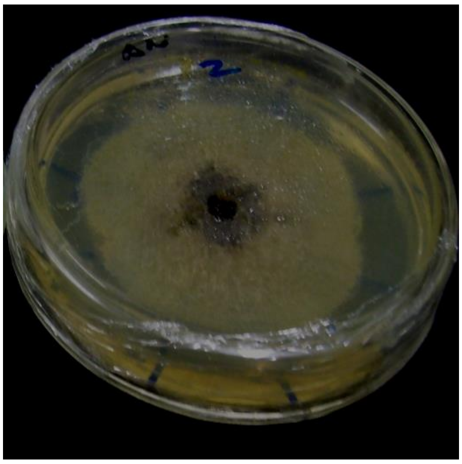

- **Figure S6**. Growth of *Botrytis cinerea* under a violet light filter.

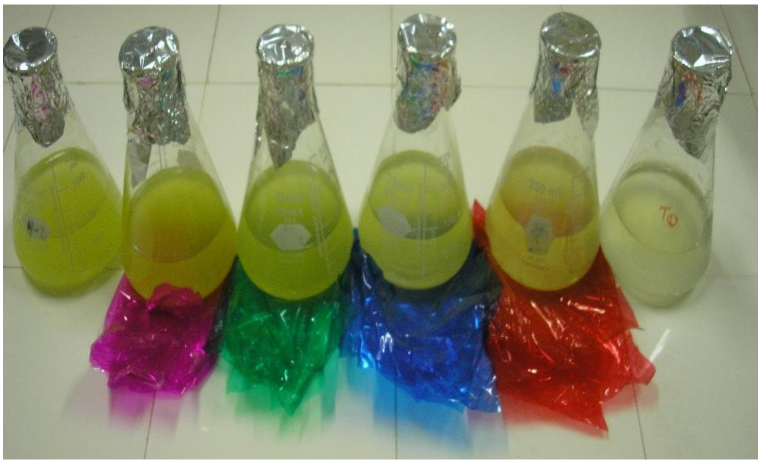

- **Figure S7**. Enzyme production by *Trichoderma harzianum* BOL-12QD under different light filters.

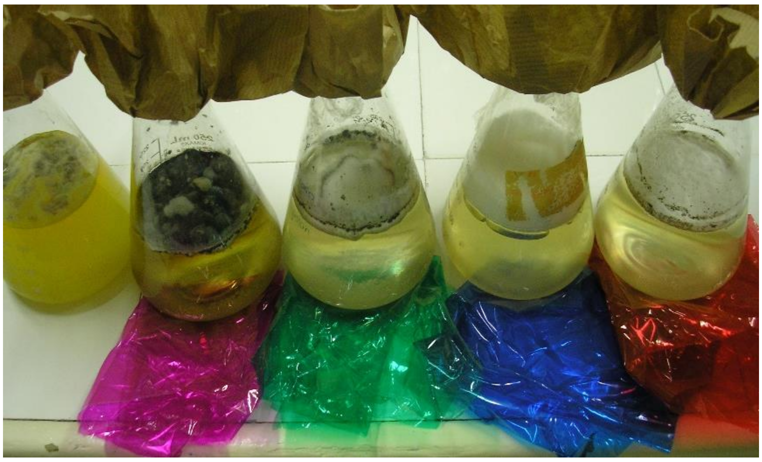

- **Figure S8**. Variability in mycelial growth and enzyme production by *Trichoderma harzianum* BOL-12QD under different light filters.

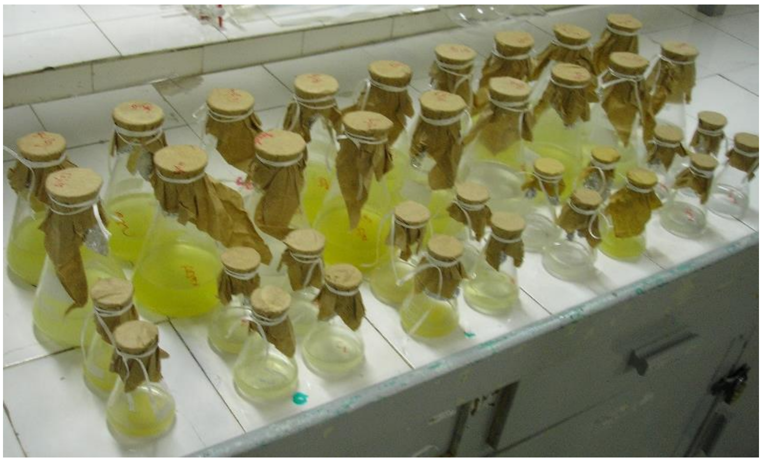

- **Figure S9**. 3^2 factorial designs: variables — potato proportion and glucose concentration.

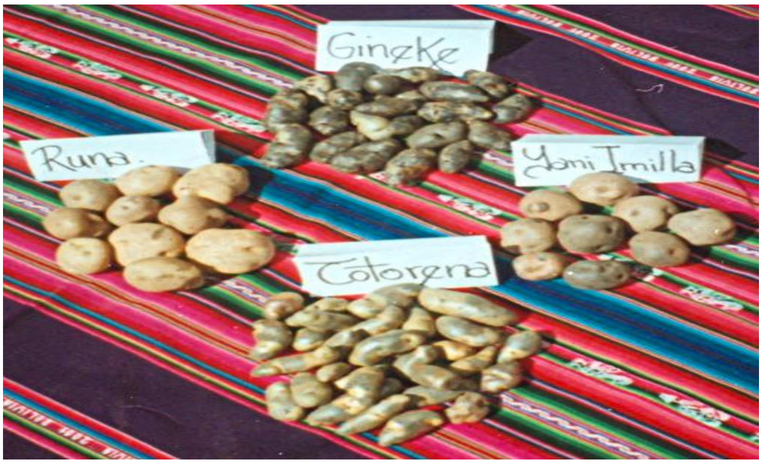

- **Figure S10**. Potato ecotypes used for enzyme production by *Trichoderma harzianum* BOL-12QD.

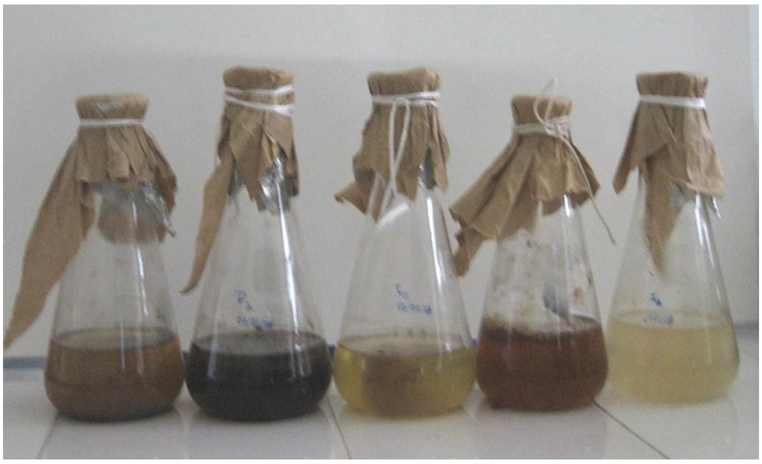

- **Figure S11**. Culture filtrates of *Trichoderma harzianum* BOL-12QD obtained using different potato ecotypes: *Condor Imilla* (*Solanum tuberosum* ssp. *andigena*), *Yana Runa* (*Solanum tuberosum* ssp. *andigena*), *Leke Pek'e* (*Solanum tuberosum* ssp. *andigena*), and *Luk'i Turno* (*Solanum* × *juzepczukii*).

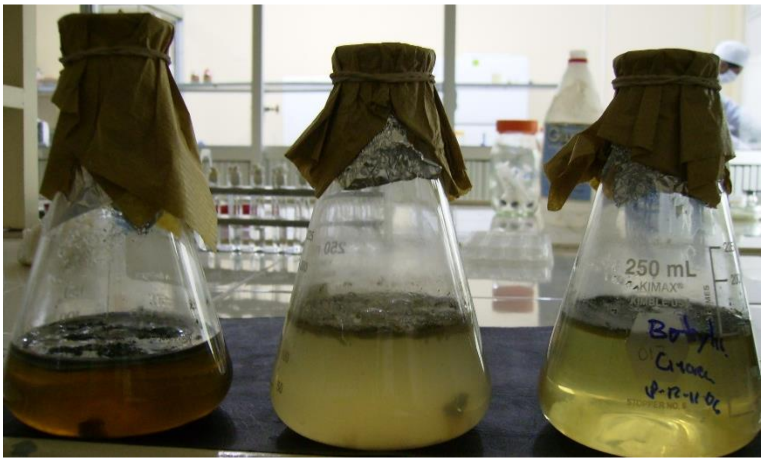

- **Figure S12**. Co-culture: culture filtrates of *Trichoderma harzianum* BOL-12QD, *Botrytis cinerea*, and their co-culture.

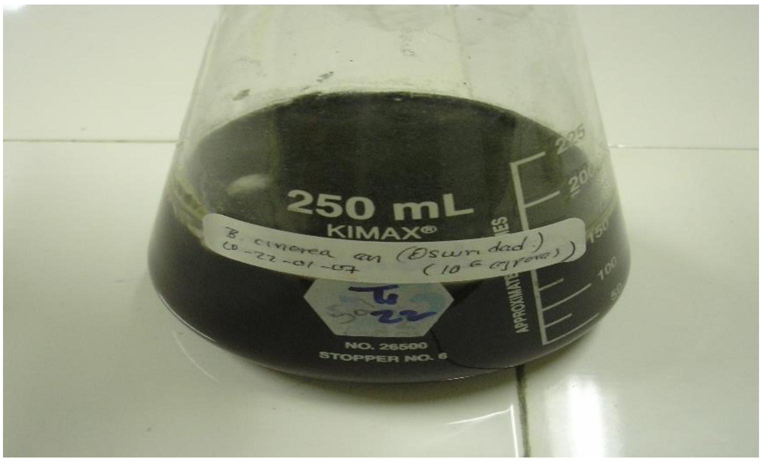

- **Figure S13**. Culture filtrate of *Botrytis cinerea*.

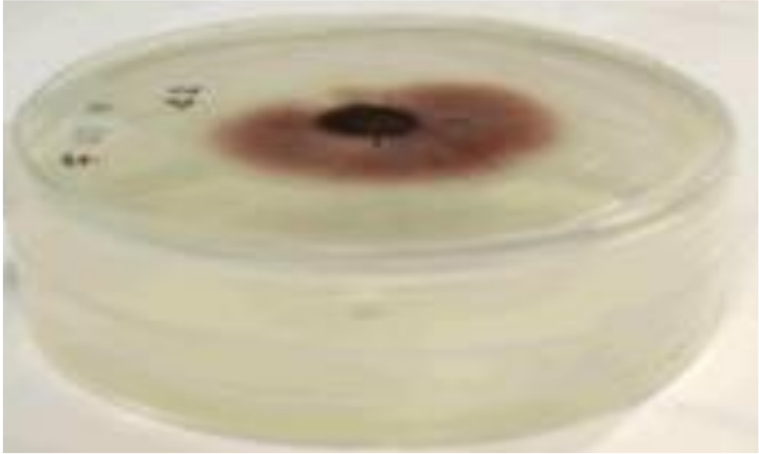

- **Figure S14**. Inhibition of *Botrytis cinerea* growth by volatile organic compounds (VOCs) produced by *Trichoderma harzianum* BOL-12QD.

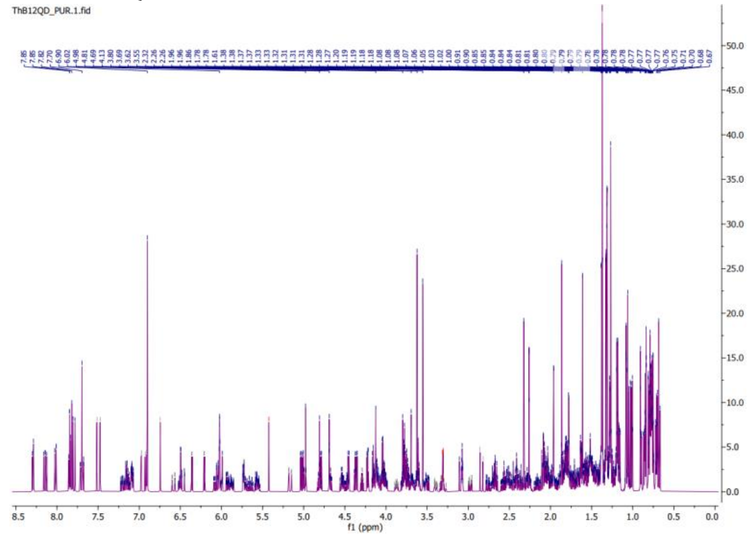

- **Figure S15**. ^{1}H NMR spectrum (500 MHz, CD_3OD) of the culture filtrate of *Trichoderma harzianum* BOL-12QD grown under violet light filter.

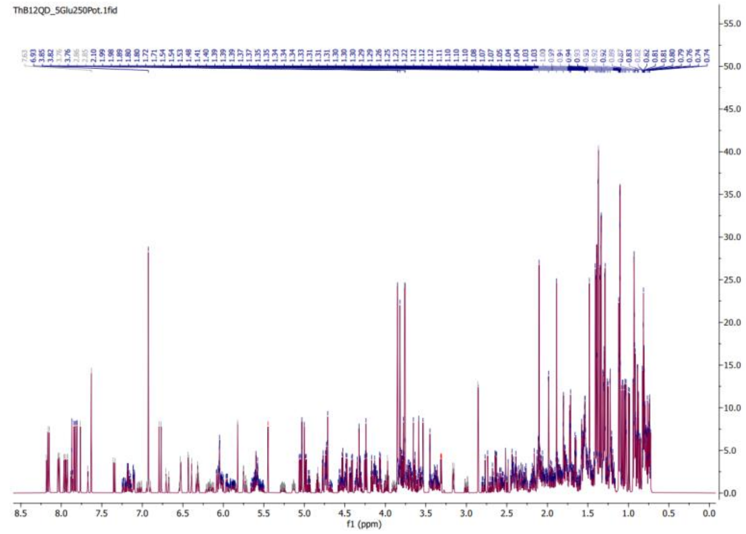

- **Figure S16**. ^{1}H NMR spectrum (500 MHz, CD_3OD) of the culture filtrate of *Trichoderma harzianum* BOL-12QD grown in a medium containing 5 g glucose and 250 g potato.

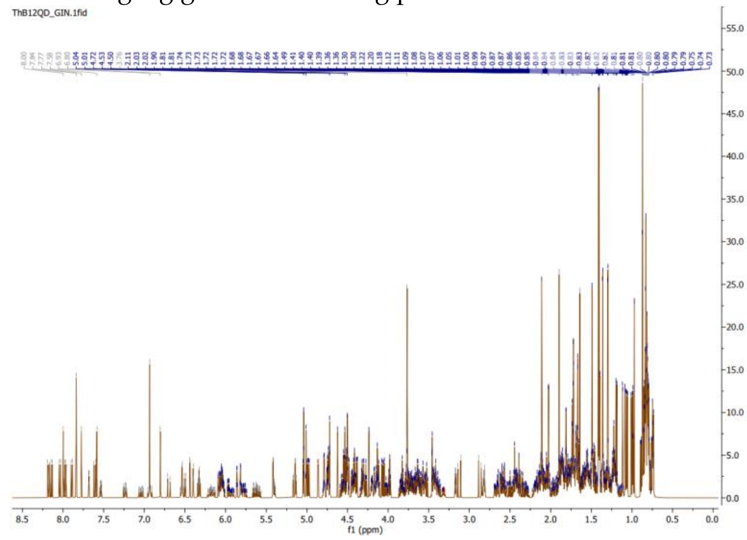

- **Figure S17**. ^{1}H NMR spectrum (500 MHz, CD_3OD) of the culture filtrate of *Trichoderma harzianum* BOL-12QD grown in a potato medium using the *Leke Pek'e* cultivar.

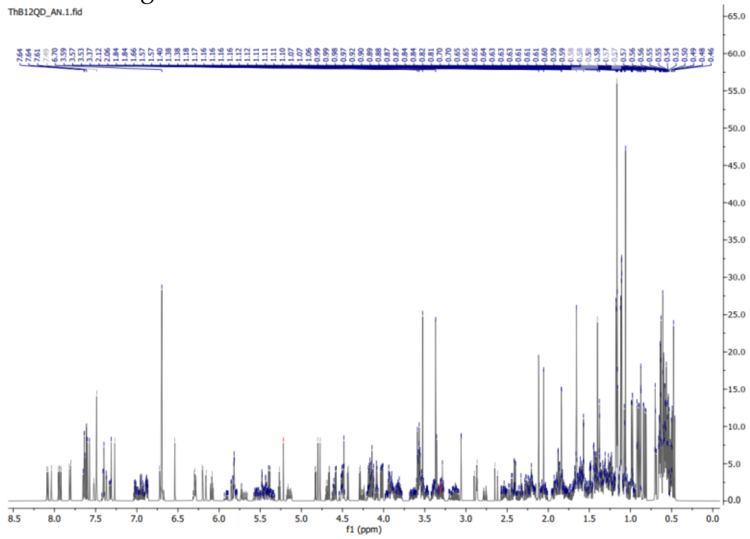

- **Figure S18**. ^{1}H NMR spectrum (500 MHz, CD_3OD) of the culture filtrate of *Trichoderma harzianum* BOL-12QD grown in a potato medium supplemented with ammonium nitrate as the nitrogen source.

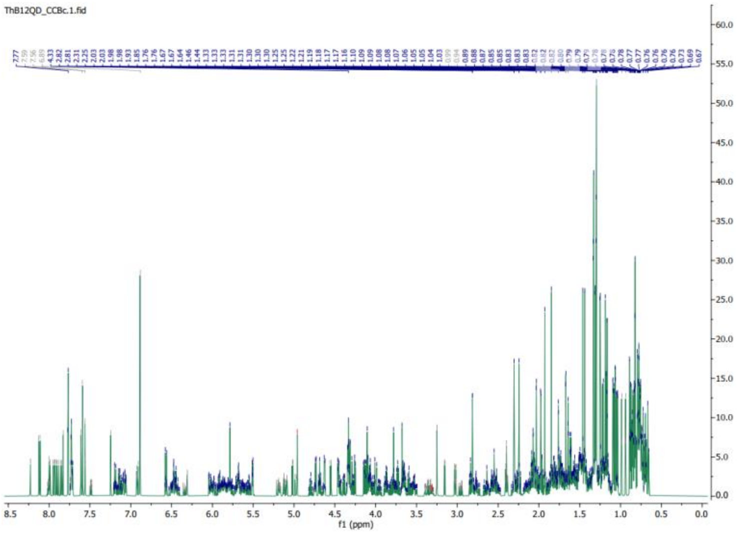

- **Figure S19**. ^{1}H NMR spectrum (500 MHz, CD_3OD) of the culture filtrate of *Trichoderma harzianum* BOL-12QD in co-culture with *Botrytis cinerea* using a 10^4 conidia mL^{-1} suspension.

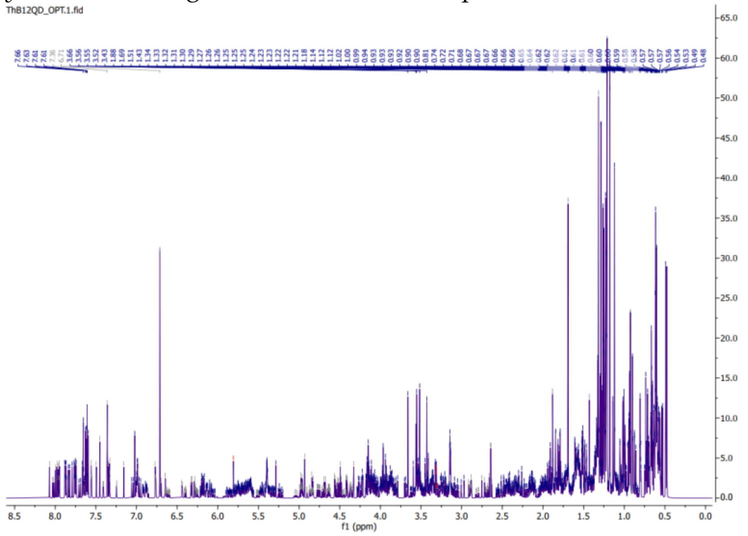

- **Figure S20**. ^{1}H NMR spectrum (500 MHz, CD_3OD) of the optimised culture filtrate of *Trichoderma harzianum* BOL-12QD grown under violet light in a medium containing 5 g glucose and 250 g potato (Leke Pek'e cultivar), supplemented with ammonium nitrate, and in co-culture with *Botrytis cinerea* using a 10^4 conidia mL^{-1} suspension.
